# Quality and utilization patterns of maternity waiting homes at referral facilities in rural Zambia: A mixed-methods multiple case analysis of intervention and standard of care sites

**DOI:** 10.1371/journal.pone.0225523

**Published:** 2019-11-27

**Authors:** Rachael Bonawitz, Kathleen L. McGlasson, Jeanette L. Kaiser, Thandiwe Ngoma, Rachel M. Fong, Godfrey Biemba, Misheck Bwalya, Davidson H. Hamer, Nancy A. Scott

**Affiliations:** 1 Department of Global Health, Boston University School of Public Health, Boston, MA, United States of America; 2 Division of Hospital Medicine, Saint Christopher’s Hospital for Children, Philadelphia PA, United States of America; 3 Department of Pediatrics, Drexel University College of Medicine, Philadelphia, PA, United States of America; 4 Department of Research, Right to Care Zambia, Lusaka, Zambia; 5 National Health Research Authority, Pediatric Centre of Excellence, Lusaka, Zambia; 6 Section of Infectious Diseases, Department of Medicine, Boston Medical Center, Boston, MA, United States of America; African Population and Health Research Center, KENYA

## Abstract

**Introduction:**

Maternity waiting homes, defined as residential lodging near a health facility, are recommended by the WHO. An improved MWH model, responsive to community standards for functionality and comfort, was implemented at two purposively selected health facilities in rural Zambia providing comprehensive emergency obstetric and neonatal care (CEmONC) services (intervention MWHs), and compared to three existing standard-of-care MWHs (comparison MWHs) at other CEmONC sites in the same districts.

**Methods:**

We used a mixed-methods time-series design for this analysis. Quantitative data including MWH quality, MWH utilization, and demographics of women utilizing MWHs were collected from September 2016 through May 2018 to capture pre-post intervention trends. Qualitative data were obtained from two focus group discussions conducted with pregnant women at intervention MWHs in August 2017 and May 2018. The primary outcomes were quality scoring of the MWHs and maternal utilization of the MWHs.

**Results:**

MWH quality was similar at all sites during the pre-intervention time period, with a significant change in overall quality scores between intervention (mean score 83.8, SD 12) and comparison (mean score 43.1, SD 10.2) sites after the intervention (p <0.0001). Women utilizing intervention and comparison MWHs at all time points had very similar demographics. After implementation of the intervention, there were marked increases in MWH utilization at both intervention and comparison sites, with a greater percentage increase at one of two intervention sites.

**Conclusions:**

An improved MWH model can result in measurably improved quality scores for MWHs, and can result in increased utilization of MWHs at rural CEmONC facilities. MWHs are part of the infrastructure that might be needed for health systems to provide high quality “right place” maternal care in rural settings.

## Introduction

There has been meaningful reduction in global maternal mortality in recent decades, with an estimated maternal mortality ratio (MMR) of 216 deaths per 100,000 live births in 2015, decreased from 385 deaths per 100,000 live births in 1990 [1]. The World Health Organization (WHO) recommends skilled care at every birth and access to facilities with emergency obstetric and neonatal care capacity to prevent maternal and infant deaths [2]. Delivery at a facility equipped to provide either basic emergency obstetric and neonatal care (BEmONC) or comprehensive emergency obstetric and neonatal care (CEmONC) has been associated with improved maternal and infant health outcomes (Table 1) [3]. However, barriers to women’s utilization of health facilities persist. Maternal factors such as education, socioeconomic status, and parity have been identified as obstacles to facility delivery [4]. Cost, transport, and distance have also been identified as barriers to facility delivery in rural settings [4–11].

**Table 1:** Summary of signal functions for basic and comprehensive emergency obstetric care as defined by WHO [3].

Basic Services	Comprehensive Services
1. Administer parental antibiotics	Perform signal functions 1-7, plus:
2. Adminster uterotonic drugs (ie, parenteral oxytocin)	8. Perform surgery (eg caesarean section)
3. Administer parenteral anticonvulsants for pre-eclampsia and eclampsia (ie, magnesium sulfate)	9. Perform blood transfusion
4. Manually remove the placenta	
5. Remove retained products (eg, manual vacuum extraction, dilation and curettage)	
6. Perform assisted vaginal delivery (eg, vacuum extraction, forceps delivery)	
7. Perform basic neonatal resuscitation (eg, with bag and mask)	

Maternity waiting homes (MWHs), defined as residential lodging near a health facility, are an intervention to decrease delays in reaching and accessing maternal care, and are recommended by the WHO [12]. While the effectiveness of MWHs on utilization of health facilities for delivery is still unclear [13–14], evidence from Zambia suggests women are more likely to deliver at a rural health facility with a MWH [8,15]. In 2014 prior to this study, Zambia had an estimated MMR of 398 per 100,000 live births, and approximately only half of women living in rural settings delivered at a health facility [16]. An improved MWH model is currently being implemented at rural BEmONC facilities and evaluated for impact on facility delivery rates in rural Zambia [17]. As part of that evaluation, new MWHs were also constructed at two purposively selected CEmONC facilities in Southern and Eastern Provinces of Zambia. Upon completion of construction, the CEmoNC facilities were provided general guidance for operations and management and assumed responsibility of the new MWHs sites. This paper uses quantitative and qualitative process evaluation data to describe the quality of implementation at intervention sites compared to existing MWHs at other CEmONC facilities in the same districts and to describe the utilization patterns of these intervention and comparison CEmONC MWHs over time.

## Methods

### Study setting

Southern and Eastern Provinces, Zambia are primarily rural. This study was nested within a larger study evaluating the effectiveness of newly-constructed, community-informed MWHs to increase access to delivery services for women living furthest from care at BEmONC sites [17]. Additionally, five CEmONC facilities within two hours’ drive time from the 10 intervention BEmONC facilities were purposively selected to be included in this study, with intervention MWHs constructed at two of those five CEmONC facilities (Table 2).

**Table 2:** Brief description of all study sites, including standard of care MWHs and newly constructed MWHs where applicable.

Study Site	Province	Facility Type	Facility Location	Average Monthly Deliveries*	Description		Personnel responsible for MWH register
					Pre-Intervention Period	Post-Intervention Period	
**Comparison Sites**							
Choma General Hospital	Southern	Government-run Level 2 Hospital	Urban	137.6	A relatives’ shelter exists where women awaiting delivery stay alongside men and women who are assisting their inpatient relatives. The building is two-room cement structure with an iron roof and missing window panes. Individuals sleep on the floor as the shelter has no beds or mattresses. Women cook with open fires on the porch immediately in front of the shelter doors. There is nearby water access with latrines, from which women also bathe.		Outpatient Health Facility staff
Kalomo District Hospital	Southern	Government-run Level 1 Hospital changed to Urban Health Center in March 2018	Urban	129.6	A large two-room relatives’ shelter exists where women awaiting delivery stay alongside men and women who are assisting inpatient relatives. The cement structure has an iron room, missing window panes, and no beds or mattresses, so men and women sleep on the cement floors. Women cook outside or in a small covered open-air cooking space next door. A large pile of debris sits a few meters from the shelter. There are latrines and a water source nearby.		Hospital Staff
Macha Mission Hospital	Southern	Mission- run Level 1 Hospital	Rural, ~70km from nearest urban center	182.9	A series of relatives’ shelters exist behind the CEmONC wards where women awaiting delivery stay alongside individuals assisting their inpatient relatives. The buildings are large, multi-room mud-brick or cement structures with three to four walls and iron roofs. Individuals sleep on the cement floors inside or on dirt outside. The complex is known as “the fires” locally for all the groups of individuals sleeping in tents or in the open and cooking during the day with small charcoal fires. There is access to a few mud-brick latrines as well as a borehole for water.		Community Member(s) residing near health facility
**Intervention Sites**							
Nyimba District Hospital	Eastern	Government-run Level 1 Hospital changed to Urban Health Center in early 2017, however, UHC remains the primary obstetric referral center for the district during study period	Urban	144.9	A two-room, cement relatives’ shelter exists where women awaiting delivery stay alongside men and women who are assisting their inpatient relatives. There are a few metal bedframes with old foam mattresses, a small covered cooking space, a few latrines and a water source nearby.	A new MWH was built specifically catering to delivering and postnatal women. The new MWH includes one large sleeping space for pregnant women for a total of 14 beds, along with one smaller room prioritizing postnatal women with four beds. Each bed has an additional mattress underneath, so the shelter can accommodate a total of 36 women. The new MWH includes bedding, electric lighting, running water, latrines, separate bathing areas, and a separate cooking shelter. Women’s companions frequently have a bed or floor mattress.	Delivery Ward Nursing Staff
Zimba Mission Hospital	Southern	Mission-run Level 1 Hospital, referral site for other hospitals	Rural, ~60km from nearest urban center	139.8	A two-room, cement, iron-roofed relatives’ shelter exists where women who are awaiting delivery stay alongside the men and women who are assisting their inpatient relatives. The building is missing window panes and has no beds or mattresses. Individuals sleep on the cement floors inside or outside in the cooking area. Women cook with open fires on the porch immediately in front of the shelter doors. There is nearby water access with latrines, from which women also bathe.	A new MWH was built specifically catering to delivering and postnatal women. The new MWH includes two large sleeping spaces for pregnant women for a total of 23 beds, along with two smaller rooms prioritizing postnatal women with five and seven beds each. Each bed has an additional mattress underneath, so the shelter can accommodate a total of 70 women. The new MWH includes bedding, electric lighting, running water, latrines, separate bathing areas, space for drying clothes, and a separate cooking shelter. Women’s companions frequently share a bed with a waiting woman or sleep in the relatives’ shelter. The old shelter continues to be used by relatives and waiting women. When a bed becomes available in the new MWH, a waiting woman transfers.	Health Facility Security Guards

A Core Maternity Waiting Home Model (Core MWH Model) was implemented at two referral hospitals (intervention CEmONC sites) capable of conducting eight or more CEmONC signal functions in Southern and Eastern Provinces. The core pillars of the Core MWH Model for CEmONC sites, derived from original formative research [18–20], include: (1) infrastructure, equipment, and supplies to address the need for higher quality, safer MWHs where women can wait comfortably for delivery; and (2) health system linkages to ensure women receive appropriate antenatal or postnatal care while waiting. The first domain encompasses the construction of a quality cement structure without leaks; a lighting source; lockable doors and windows; a cooking area with utensils; bathing and laundry areas; latrines; beds, bedding, and mosquito nets; a lockable storage room for assets; dedicated space for postnatal women and newborns to stay; and access to water for drinking and hygiene [17]. The second domain requires being adjacent to a CEmONC facility and for CEmONC staff to regularly monitor waiting women and the condition of the MWH. Immediately after construction of the Core MWH Model at the intervention sites, ongoing maintenance of the MWH was assumed by the affiliated hospitals. ‘Policies, management and finance’ was a third pillar included in the main evaluation, but was not included in the CEMONC implementation plan. Upon completion of construction, the CEMONC sites assumed responsibility of the MWHs, with minimal guidance around management and governance.

Three comparison CEmONC sites in Southern Province continued implementing the MWH ‘standard of care,’ which varied in quality (Table 2). A register system was instituted at all sites to capture MWH utilization (S1 File). A designated person who received a small stipend completed the registers at each site (Table 2).

Intervention site Zimba opened in March 2017 and Intervention site Nyimba opened in April 2017. During construction of the MWHs in early 2017, Nyimba was re-designated an urban health center from a Level 1 Hospital. However, maternity services (including CEmONC signal functions) remained at Nyimba, and Nyimba remained the primary obstetric referral center for its district during the course of this evaluation. Similarly, Kalomo changed from a Level 1 Hospital to an urban health center in March 2018 and all CEmONC functions were transitioned to the new hospital a month later (Table 2).

At Zimba, two shelters exist: the Core MWH Model and the prior existing MWH which accommodates any overflow. If a waiting woman transfers from the old MWH to the Core MWH Model, she is not re-registered, and the time spent at each MWH is not known. Data for Zimba thus include women waiting at the existing MWH and the Core MWH Model. Each woman is counted only once at initial registration (Table 2).

### Study design and methods

We used an interrupted, two-group time-series design, systematically assessing the two intervention sites (Core MWH Model) and three comparison sites (standard of care) on a monthly basis between September 2016 and May 2018. We define the pre-intervention period to be from September 2016 through the opening of each MWH intervention site (March or April 2017) and a post-intervention period to be the 14 months following the opening of each site, through May 2018.

We used mixed-methods to capture and triangulate data. First, quality assessment data were collected monthly from both intervention and comparison sites using a quantitative core model checklist (CMC), which was developed specifically for this project to measure quality, implementation fidelity and maintenance of quality after implementation (S2 File). This CMC evaluated nine core quality components of the MWHs identified during formative research [18–20]: infrastructure, safety, amenities, cleanliness, water, hygiene, sanitation, cooking and feedback. Second, MWH utilization data were extracted monthly from both intervention and comparison sites. Registers captured individual-level demographics and MWH arrival and discharge dates. We did not calculate a sample size for utilization *a priori* as these data were collected as part of routine monitoring. Local data collectors who underwent ethics training and training in all study instruments completed register data extraction and quality assessment data collection. Third, we conducted two focus group discussions (FGD) with sixteen pregnant women at each intervention MWH (S3 File) after the MWHs had opened. These four FGDs, facilitated by local data collectors trained in research ethics, qualitative interviewing techniques, the specific instruments, and fluent in the relevant local languages, captured perspectives on MWH quality, barriers and facilitators to MWH access and facility delivery, and reasons for MWH use. Women 15 years or older who had been staying the longest at the MWHs were recruited to participate in FGDs.

### Study variables

Primary quantitative outcomes for this analysis are quality scoring of CEmONC facilities and utilization of MWHs at CEmONC facilities. To construct the composite quality score, the following domains were systematically assessed via the CMC: infrastructure, safety, amenities (including bedframes, mattresses, mosquito nets), cleanliness, water (access to potable water), hygiene (bathing area), sanitation (latrines), cooking (designated area and utensils), and feedback (system for receiving and addressing women’s comments/complaints). Each domain had between one to ten core components; the presence or absence of any individual component was scored as one or zero respectively. If present, additional points, if applicable, were added depending on material type (e.g. metal vs. thatched roof), functionality (e.g. absence of holes or leaks in roof), quantity of non-broken assets (e.g. bedframes), and condition (e.g. cleanliness scored as sufficient, needs improvement, or not clean). Scores under each domain were summed and standardized to 10. The domains were then summed to create a monthly composite quality score and scaled to 100.

Indicators of utilization included mean number of women staying per month, average daily census (ADC), bed occupancy rate (BOR), and average length of stay (ALOS). Women were categorized as either staying for less than one night in the MWH or at least one night. The mean number of women utilizing an MWH per month was calculated by summing the total number of women who stayed at the MWH for any amount of time for any reason. Women who stayed less than one night, and/or had missing discharge and delivery dates were included in the mean number of women staying per month but excluded from calculations for average daily census (ADC), bed occupancy rate (BOR), and average length of stay (ALOS). ADC was calculated by summing total bed-days for all women who stayed at the MWH each month divided by the number of days in the month. Bed occupancy rate is ADC divided by the number of beds multiplied by one hundred. The ALOS was calculated by summing the bed-days for all women staying at the MWH divided by the number of women. For all variables, a woman’s contribution was counted for the calendar month in which she arrived at the MWH.

Demographic characteristics include age, grade level completed, marital status, gravida, parity, pervious stillbirths, gestational age (EDD as reported on the ANC card on admission to the MWH), travel time from home, transportation mode, number of companions with the woman, and the companions’ relationship. Demographics are reported on all women who utilized any MWH for any length of time (including those without a discharge date).

### Data management and analysis

For the quality scores we calculated the mean and standard deviations. We used a difference-in-differences (DID) analysis to test for significance in quality between intervention and comparison sites during the pre- and post-intervention periods. The composite score and scores for individual domains are reported.

We tested for significance in all quantitative data using first a t-test or chi-squared test for differences between intervention and comparison sites during the pre-intervention time period, and then a DID analyses for the post-intervention time period at the intervention sites as comparison sites lacked beds. For mean quality assessment scores, mean number of women per month, and ADC, the difference-in-difference estimates controlled for month due to the monthly nature of these variables. All analyses accounted for clustering. BOR was only calculated for the post-intervention period. Utilization data are presented as aggregate and stratified by intervention site; the two intervention sites had different pre-intervention utilization patterns. All quantitative analysis was done using SAS 9.4 (Cary, NC). P-values were considered significant at a level of alpha≤0.05.

All qualitative analysis was conducted using NVivo 11 Software (QSR International, Doncaster, Australia). The FGDs were audio recorded, translated and transcribed verbatim into English. Some codes were created *a priori* based on the FGD guide; additional codes were created as themes emerged during the coding process. A content analysis was done for each time point and then the emerging themes were compared over time [21]. Qualitative data were triangulated with quantitative data to create a full picture of MWH quality and choice to utilize MWHs.

### Ethical review

Ethical approval was granted by the Boston University Institutional Review Board (protocol H-35321) and the ERES Converge IRB in Zambia (reference number 2016-June-023). Approval was also obtained from the Zambia National Health Research Authority and the Ministry of Health. The hospital administrator at each CEmONC site also granted approval for the evaluation. Written informed consent was obtained from each FGD participant. For FGD participants aged 15-17, assent was first obtained from the woman and consent was obtained from her guardian. If no guardian was available, the woman was ineligible for participation in the FGD. A waiver of consent was granted for data extracted from the registers. Quality assessment of the MWHs using the Core Model Checklist was non-human subjects research.

## Results

We first present the FGD participant demographics because supporting qualitative data are threaded throughout the results section. We then present the quality assessment results followed by the utilization patterns at both intervention and comparison MWHs.

### FGD participant demographics

All FGD participants were pregnant women waiting to deliver. The average woman was aged 24.5 years (SD 6.1 years), had some schooling (average grade level completed 2.9, SD 3.9 levels), was married (84.4%), and had been pregnant before. The demographics between women who participated in FGDs at Nyimba and Zimba were similar (Table 3).

**Table 3:** Demographics of focus group discussion participants at intervention maternity waiting homes.

	Overall	Nyimba	Zimba
	N = 32	N = 16	N = 16
Age, mean (SD)	24.5 (6.1)	22.4 (5.6)	26.5 (6.0)
Highest grade completed, mean (SD)	2.9 (3.9)	2.0 (3.1)	3.9 (4.5)
Marital status, N (%)			
*Married/cohabitating*	27 (84.4)	12 (75.0)	15 (93.7)
*Divorced/separated/widowed*	0 (0.0)	0 (0.0)	0 (0.0)
*Never married*	5 (15.6)	4 (25.0)	1 (6.3)
Gestational age (months), mean (SD)	8.9 (0.2)	8.9 (0.3)	9.0 (0)
Previous pregnancies, mean (SD)	2.9 (2.3)	2.4 (2.1)	3.4 (2.4)
Number of live children, mean (SD)	1.8 (2.2)	1.4 (2.0)	2.3 (2.3)

SD = standard deviation

### MWH quality assessment

The quality of all CEmONC MWHs was similar during the pre-intervention period; the mean composite quality score was 38.7 (SD 7.9) out of 100 for comparison sites and 44.5 (SD 7.6) out of 100 for intervention sites (p = 0.260) (Table 4). Only the hygiene and amenities scores were significantly different between intervention and comparison sites at baseline. We observed a significant change in the composite quality score between intervention and comparison sites post-intervention (p = 0.008). Nearly all individual domains were significantly better at intervention sites during the post-intervention period, though no difference was observed in the cooking score (p = 0.405). The largest differences were seen in the scores for sanitation (DID estimate 5.3, p<0.001), safety (DID estimate 6.3, p<0.001), and amenities (DID estimate 6.7, p<0.001).

**Table 4:** Monthly MWH quality assessment scores by pre/post time periods and study arm.

	Pre-Intervention		Post-Intervention		Pre-Pre P-Value	DID Estimate	DID P-Value
	September 2016 - February 2017		March 2017*- May 2018				
	Comparison	Intervention	Comparison	Intervention			
	Mean (SD)	Mean (SD)	Mean (SD)	Mean (SD)			
Composite Score**	38.7 (7.9)	44.5 (7.6)	43.1 (10.2)	83.8 (12.0)	0.260	34.2	0.008
*Infrastructure Score^*	7.6 (1.8)	7.6 (2.0)	6.5 (1.3)	9.2 (1.0)	0.836	2.6	0.004
*Water Score^*	10 (0)	10 (0)	8.4 (3.7)	10 (0)	-	1.6	0.009
*Sanitation Score^*	2.4 (2.5)	2.8 (2.5)	2.3 (2.6)	7.7 (2.3)	0.872	5.3	<0.001
*Hygiene Score^*	2.1 (1.1)	3.7 (1.7)	3.3 (2.3)	7.3 (2.9)	0.074	2.7	0.011
*Cooking Score^*	6.2 (5.1)	8.0 (4.2)	7.8 (3.8)	8.4 (2.1)	0.175	-1.2	0.405
*Safety Score^*	2.0 (0.7)	2.5 (1.4)	2.3 (1.2)	8.8 (1.5)	0.727	6.3	<0.001
*Clealiness Score^*	2.0 (2.5)	2.7 (2.6)	1.6 (2.6)	5.9 (4.2)	0.633	3.8	0.006
*Amenities Score^*	0	3.0 (2.5)	0.1 (0.3)	9.5 (0.8)	0.009	6.7	<0.001
*Feedback Score**^*	0.6 (2.4)	0	5.8 (5.0)	8.6 (3.5)	0.361	3.26	0.005

DID = difference-in-differences; SD = standard deviation

*One intervention site has pre-intervention from September 2016 through March 2017, and post-intervention data from April 2017 through May 2018 due to the site opening in April 2017.

**The composite score is the sum of all scores, out of 90. Scaled to be out of 100

^ Scored out of 10

FGD results corroborate the quality score patterns. Women were generally satisfied with the quality of the newly-constructed MWHs and appreciated having available amenities including beds, mattresses, blankets, mosquito nets, and cooking utensils.

“It is the first time for me to come here so I was not confident, as it is not easy to carry a mattress when the vehicle is not yours. I was thinking that I was coming here to suffer, but it was the opposite, I was very surprised to see these beds.” – Waiting woman, Zimba

Similar to the quantitative findings, women identified inadequate storage space for their food. Even after implementation at the intervention sites, women complained that the cooking area was too small and dirty:

“The place where we cook from is dirty. It’s windy. So when we start cooking and there is a blast of wind, dirt goes into the food.” – Waiting woman, Nyimba

“The place is very small, because we are many. Where we cook from, sometimes you may even step on your friend’s pot. The place we cook from is too small.” – Waiting woman, Zimba

### MWH utilization assessment

#### Demographics of MWH users

Women utilizing MWHs at all intervention and comparison sites were similar during the pre- and post-intervention periods (Table 5). Overall, waiting women at either intervention or comparison sites had a mean age of approximately 25 years, with a third of women 19 years or younger, and about 20% 35 years or older. Approximately a third of women were experiencing their first pregnancy, and 30% had more than five previous pregnancies. Women arrived at the MWH at approximately their 38th week of pregnancy based on their estimated delivery date. The vast majority of women were married and had completed a mean of 6 years of schooling.

**Table 5:** Demographics of women utilizing any CEmONC MWHs by time period and study arm^.

	Pre-Intervention		Post-Intervention		Pre/Pre P-value	DID Estimate	DID P-Value
	September 2016 – February 2017		March 2017*- May 2018				
	Comparison N = 719	Intervention N = 371	Comparison N = 1977	Intervention N = 2796			
Age in years, mean (SD)	24.8 (8.3)	25.5 (8.3)	25.4 (8.5)	26.5 (8.5)	0.349	0.5	0.477
Age categories, N (%)							
Under 15	8 (1.3)	1 (0.3)	27 (1.4)	17 (0.6)	0.620	0	0.382
15–19	236 (38.2)	113 (32.7)	700 (27.2)	766 (28.4)			
20–24	107 (17.3)	74 (21.4)	333 (17.0)	548 (20.3)			
25–29	76 (12.3)	64 (18.6)	214 (10.9)	364 (13.5)			
30–34	81 (13.1)	30 (8.7)	279 (14.2)	349 (12.9)			
35 +	110 (17.8)	63 (18.3)	402 (20.7)	649 (24.1)			
Gravida, mean (SD)	3.4 (3.0)	3.7 (2.8)	4.0 (3.0)	4.1 (2.9)	0.479	-0.2	0.746
Primagravida, N (%)	302 (43.0)	107 (30.2)	694 (35.3)	770 (28.5)	0.093	0	0.315
Parity, mean (SD)	2.7 (2.9)	2.4 (2.5)	3.1 (2.9)	2.7 (2.6)	0.367	-0.1	0.792
Grand multipara (parity≥5), N (%)	185 (25.7)	71 (19.1)	677 (34.2)	760 (27.2)	0.406	0	0.864
Women with at least one previous stillbirth, N (%)	86 (12.0)	75 (20.2)	323 (16.3)	771 (27.6)	0.209	0	0.895
Number of previous stillbirths, mean (SD)	0.1 (0.5)	0.3 (0.6)	0.1 (0.5)	0.4 (0.9)	0.120	0.1	0.358
Gestational age upon arrival at MWH (weeks), mean (SD)**	38.4 (2.5)	38.3 (2.4)	38.2 (2.3)	38.6 (2.7)	0.795	0.5	0.268
Marital status, N (%)							
Married/cohabitating	590 (82.5)	276 (89.3)	1564 (79.5)	2389 (88.4)	0.20	0	0.732
Divorced/separated/widowed	7 (1.0)	11 (3.5)	23 (1.2)	27 (1.0)			
Never-married	118 (16.5)	22 (7.1)	380 (19.3)	286 (10.6)			
Highest grade completed, mean (SD)	6.6 (2.2)	6.1 (2.9)	7.0 (2.1)	6.2 (2.9)	0.325	-0.3	0.090
Transport used to MWH, N (%)							
Walking	45 (6.4)	10 (2.9)	100 (5.1)	121 (4.5)	0.669	0	0.045
Bicycle	46 (6.5)	8 (2.3)	126 (6.4)	42 (1.6)			
Wheelbarrow / Ox-cart	14 (2.0)	3 (0.9)	37 (1.9)	43 (1.6)			
Taxi / Car / Motorcycle	591 (84.4)	316 (91.9)	1692 (86.2)	2305 (85.7)			
Ambulance	4 (0.6)	7 (2.0)	8 (0.4)	180 (6.7)			
Travel time from home to MWH (hours), mean (SD)	2.8 (2.2)	3.2 (2.1)	3.3 (2.2)	3.4 (2.3)	0.431	-0.4	0.171
Number of companions per woman, mean (SD)	1.0 (0.3)	1.4 (1.0)	1.0 (0.3)	1.1 (0.5)	0.014	-0.3	0.039
Relationship of companion to waiting woman, N (%)							
Mother/Mother-in-law	429 (59.7)	229 (61.7)	1074 (54.3)	1506 (53.7)	0.730	0	0.680
Other female relative	193 (26.8)	52 (14.0)	653 (33.0)	727 (26.0)	<0.001	0.1	0.018
Child	20 (2.8)	3 (0.8)	86 (4.4)	50 (1.8)	0.254	0.1	0.186
Husband	15 (2.1)	1 (0.3)	8 (0.4)	11 (0.4)	0.264	0.5	0.005

MWH = maternity waiting home; DID = difference-in-differences; SD = standard deviation

^ This table includes all women utilizing any MWH for any amount of time, even those without known discharge dates

* One intervention site has pre-intervention from September 2016 through March 2017, and post-intervention data from April 2017 through May 2018 due to the site opening in April 2017.

** Gestational age based on estimated delivery date included on woman’s ANC card

During both the pre- or post-intervention periods, most women arrived at the MWH using motorized transport; significantly more women arrived at both intervention sites via ambulance during the post-intervention period (2.0% vs 6.7%, p = 0.045). On average, women traveled about three hours to reach the facility from their homes, largely driven by Zimba users (Zimba = 3.7 hours, Nyimba = 1.3 hours, p<0.001).

The majority of women were accompanied to the MWH by their mother, mother-in-law or another female relative. Pre-intervention, women staying at intervention sites brought significantly more companions than those staying at comparison sites (p = 0.014); however, after implementation, they brought a similar number of women as the comparison sites (DID estimate of -0.3, p = 0.0039). FGDs respondents discussed the importance of having a companion with them at the MWH:

“If you have someone to look after you here it becomes easy to use the MWH.” – Waiting woman, Nyimba

#### Total utilization

During the pre-intervention time period, significantly more women utilized the standard-of-care MWH at Zimba (N = 310) compared to Nyimba (N = 52) (p<0.0001). All pre- and post- utilization data is thus disaggregated by intervention site. After implementation of the Core MWH Model, there were marked increases in the number of women staying at the MWHs for any time at both comparison and intervention sites, with a greater increase at both intervention sites than the comparison sites (Table 6). However, over the study period, mean monthly deliveries were similar between sites (Zimba: 140, Nyimba: 145, Choma: 130, Kalomo: 138, Macha: 183) (Table 2).

**Table 6:** Patterns of utilization of MWHs at CEmONC facilities by time period and study arm.

	Pre-Intervention			Post-Intervention			Nyimba			Zimba		
	September 2016 - February 2017			March 2017*- May 2018								
	Comparison	Nyimba	Zimba	Comparison	Nyimba	Zimba	Pre-Pre P-value*	DID Estimate	DID P-value*	Pre-Pre P-value*	DID Estimate	DID P-value*
	N (%)	N (%)	N (%)	N (%)	N (%)	N (%)						
Women who used the MWH:	N = 719	N = 52	N = 310	N = 1977	N = 357	N = 2439	0.529	-0.1	0.574	0.248	-0.6	0.008
*For at least 1 night*	417 (58.0)	44 (71.1)	73 (23.6)	1460 (73.8)	311 (87.1)	2145 (87.9)						
*For less than 1 night*	302 (42.0)	17 (28.9)	237 (76.4)	517 (26.2)	46 (12.9)	294 (12.1)						
Among women who did not stay at least one night:	N = 302	N = 17	N = 237	N = 517	N = 46	N = 294	0.395	0.1	0.215	0.455	0.5	0.010
*Women have an MWH* discharge or delivery date	20 (6.6)	3 (17.6)	28 (11.8)	58 (11.2)	18 (39.1)	183 (62.2)						
*Women missing MWH* discharge or delivery date	282 (93.4)	14 (82.3)	209 (88.2)	459 (88.8)	28 (60.9)	111 (37.8)						

DID = difference-in-differences

* p-values are comparisons of the distribution of the frequency, and not categories.

FGD respondents expressed enthusiasm about staying at the Core MWH Model after having seen or heard about it:

“I am very happy I saw this house when it was being built, I was waiting for my time to come.” – Waiting woman, Zimba

For women who did not stay at the MWH for at least one night, there is a substantial amount of missing data, especially at comparison sites. Delivery or discharge dates are known for only 11% of women at comparison sites, compared to 62% at Zimba and 39% at Nyimba (Table 6).

#### Utilization patterns

**Fig 1:**
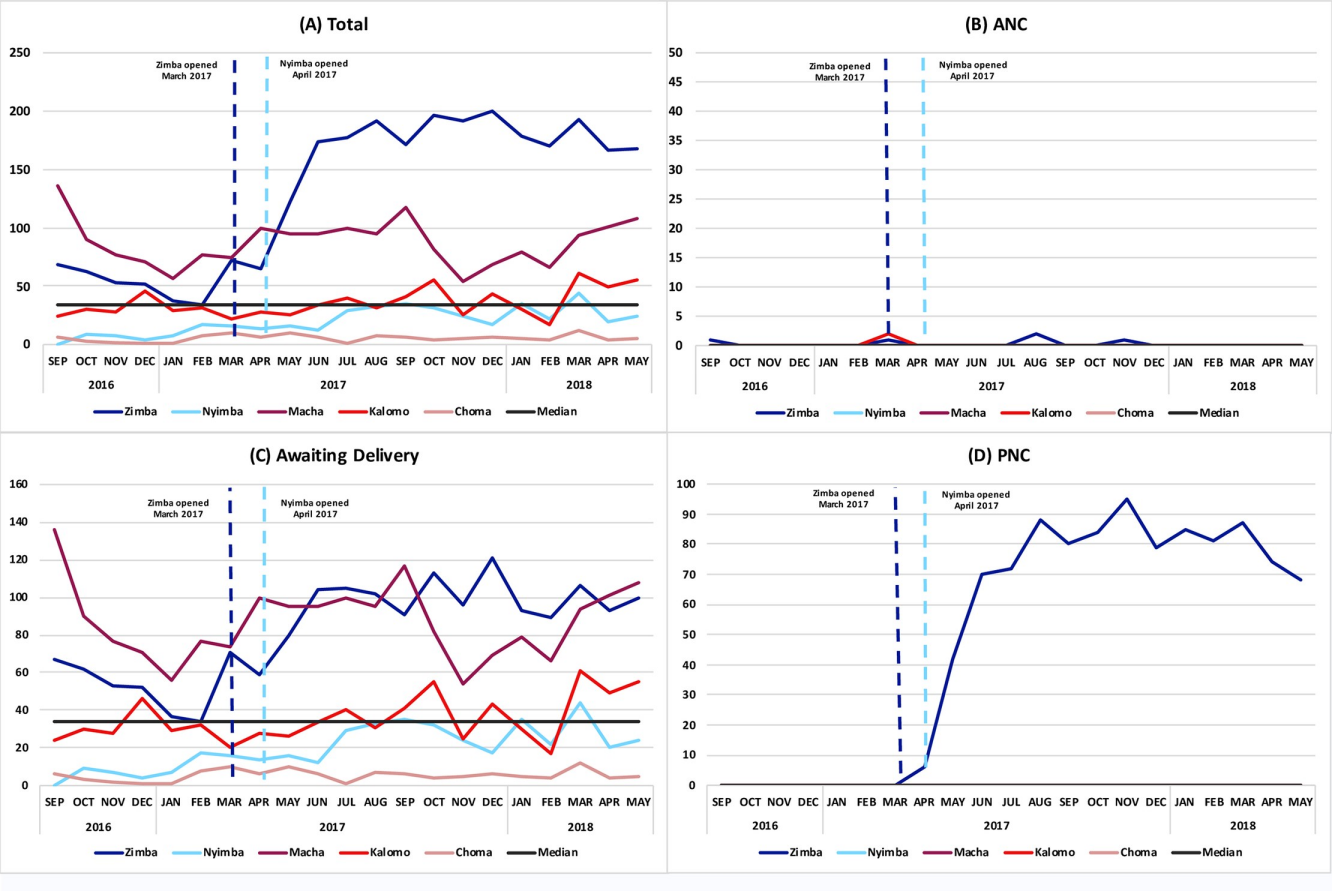
Figs depicting mean number of women utilizing MWHs per month, stratified by: any utilization (1A), utilization for ANC (1B), utilization while awaiting delivery (1C), and utilization for PNC (1D), with each MWH represented by a colored line.

Utilization of CEmONC-affiliated MWHs for any reason was consistently higher over time at intervention than at comparison sites (Fig 1A–1D), driven primarily by utilization at Zimba (Fig 1A, Table 7). Utilization of MWHs for ANC did not significantly change over time (Fig 1B), and the majority of women utilized MWHs while awaiting delivery (Fig 1C). The utilization of MWHs for PNC increased at Zimba only (Fig 1D), though both intervention sites had dedicated space for PNC users (Table 2).

**Table 7:** Maternity waiting home utilization, stratified by reason for stay, by time period and study arm.

	Pre-Intervention			Post-Intervention			Nyimba			Zimba		
	September 2016 - February 2017			March 2017* - May 2018			Pre-Pre P-Value	DID Estimate	DID P-Value	Pre-Pre P-Value	DID Estimate	DID P-Value
	Comparison	Nyimba	Zimba	Comparison	Nyimba	Zimba						
	Mean (SD)	Mean (SD)	Mean (SD)	Mean (SD)	Mean (SD)	Mean (SD)						
**Mean number of women using the MWH, monthly, by reason for stay^**												
Total	39.8 (37.9)	10.0 (5.3)	51.0 (13.4)	43.9 (36.7)	25.5 (9.4)	162.5 (42.6)	0.317	8.9	0.131	0.697	107.4	<0.001
Antenatal	0 (0)	0 (0)	0.2 (0.4)	0.0 (0.3)	0.1 (0.3)	0.3 (0.6)	-	0	0.587	<0.001	0	0.338
Awaiting Delivery	39.8 (37.9)	10.0 (5.3)	50.8 (13.2)	43.9 (36.6)	25.5 (9.4)	94.9 (15.9)	0.317	8.9	0.131	0.701	40.0	<0.001
Postnatal	0 (0)	0 (0)	0 (0)	0 (0)	0 (0)	67.4 (28.9)	-	-	-	-	67.4	<0.001
**Average daily census, by reason for stay**												
Total	11.1 (10.2)	3.3 (3.0)	5.0 (4.1)	17.3 (14.9)	11.8 (4.5)	43.9 (7.9)	0.316	2.1	0.647	0.426	32.6	0.003
Antenatal	0 (0)	0 (0)	0 (0)	0 (0)	0 (0)	0 (0)	-	0	0.819	-	0	0.009
Awaiting Delivery	11.0 (10.2)	3.3 (3.0)	5.0 (4.0)	17.3 (14.9)	11.8 (4.5)	39.3 (6.2)	0.320	2.0	0.651	0.431	28.1	0.005
Postnatal	0 (0)	0 (0)	0 (0)	0 (0)	0 (0)	4.4 (2.0)	-	-	-	-	4.4	<0.001
**Average length of stay, by reason for stay**												
Total	14.6 (13.3)	11.7 (11.3)	12.6 (15.2)	16.2 (12.8)	15.5 (13.6)	8.8 (11.4)	0.220	2.6	0.162	0.517	-5.9	0.025
Antenatal	0 (0)	0 (0)	0 (0)	1 (0)	0 (0)	1.2 (1.2)	-	-	-	-	0.25	<0.001
Awaiting Delivery	14.8 (13.2)	12.3 (11.3)	13.1 (15.3)	16.3 (12.8)	15.6 (13.6)	14.0 (12.8)	0.254	2.3	0.210	0.503	-0.6	0.687
Postnatal	0 (0)	0 (0)	0 (0)	0 (0)	0 (0)	2.0 (1.8)	-	-	-	-	2.0	<0.001

DID = difference-in-differences

^ This indicator includes all women utilizing any MWH for any amount of time, even those without known discharge dates. Average daily census and average length of stay values only include those women with a known discharge or delivery date.

*One intervention site has pre-intervention from September 2016 through March 2017, and post-intervention data from April 2017 through May 2018 due to the site opening in April 2017

There was no significant difference in mean number of women staying for any length of time and for any reason at Nyimba compared to comparison sites (DID estimate 8.9, p = 0.701). There was a marked difference in mean number of women utilizing the MWH for any length of time and for any reason at Zimba compared to comparison sites (DID estimate 107.4, p<0.001) (Table 7). Very few women overall utilized the MWHs for ANC, and only Zimba was utilized by postnatal women (DID estimate 67.4 mean women per month, p <0.001)

Qualitatively, most women stated they were referred to a CEmONC facility at ANC (presumably at a BEmONC site, though they could have received ANC at the CEmONC facility) for three main reasons: 1. Having a complication that required higher level care; 2. Being primigravida; or 3. Being grand multi-parous (>=5):

“When the doctor sends you to a big hospital it is because they have seen a problem that you have. The one who doesn’t seem to have any problems will wait at the clinic in our communities.” – Waiting woman, Nyimba

“They told me that for the first pregnancy you have to deliver from a hospital, that is why I came here.” – Waiting woman, Zimba

“This is my fifth pregnancy and we were told at the clinic that when you have your fifth pregnancy and any other succeeding ones, you need to deliver from a big hospital.” – Waiting woman, Nyimba

Average daily census (ADC) was significantly higher at Zimba after the intervention compared to comparison sites (DID estimate 32.6 women, p = 0.003), though there was no significant difference in ADC at Nyimba compared to comparison sites (DID estimate 2.1 women, p = 0.647) (Table 7). Similar results were found for the ADC of postnatal women at Zimba compared to comparison sites (DID estimate 4.4, p<0.001). Post-intervention, women stayed longer at comparison sites than either intervention site while awaiting delivery (comparison site ALOS 16.3 days, SD 12.8; Nyimba ALOS 15.6 days, SD 13.6; and Zimba ALOS 14.0 days, SD 12.8) (Table 7). Among all women waiting for any reason, ALOS was significantly lower for Zimba (DID estimate -5.9, p = 0.025) due to the large number of postnatal stays, which averaged an expected 2 days (SD 1.8) (Table 7). Bed occupancy rate for Zimba was 129% and for Nyimba was 84%.

Corroborating the quantitative utilization findings, women at intervention sites discussed the challenge of overcrowding during FGDs:

“When all the beds that are inside are filled up, we start sleeping two per bed.” – Waiting woman, Nyimba

“People are still sleeping outside. They will not even have the opportunity to enter this MWH. We are asking that you make this house bigger.” – Waiting woman, Zimba

Women at both intervention sites mentioned having to share a bed or a mattress on the floor with their companion. Over-crowdedness was especially a challenge at Zimba. If the newly constructed MWH was full, women would sleep in the old MWH or outside and move into the newly constructed MWH if space became available (Table 2).

## Discussion

After the implementation of the Core MWH Model at CEmONC facilities, the quality of those MWHs increased, and utilization of the improved MWHs increased compared to comparison sites. All MWHs were of comparable quality during the pre-intervention phase, though the intervention MWHs had higher hygiene and amenities scores at baseline. These differences in scores at baseline, however, are likely of limited significance, as the intervention MWHs were fully (re)constructed in accordance with the Core MWH Model, regardless of baseline quality (Table 4). Intervention MWHs had significant improvements in the quality scores in all domains except the cooking score. Qualitative data suggest that the Core MWH Model cooking spaces were too small for the number of women needing to access them, and were exposed to wind and dirt. There were no specifications regarding cooking space size or amenities in the Core MWH Model, only the requirement that there be a covered space for cooking from formative research conducted prior to implementation [20]. Additionally, despite intensive planning and thoughtful projections for bed numbers needed, the Core MWH Model sites were still overcrowded, particularly Zimba (BOR of 129%), which inherently compromises perceived quality (a bed occupancy rate not exceeding 100% ensures that there is not more than one woman per bed).

While our results show that an improved MWH model can result in measurably improved quality across multiple domains, there are likely additional drivers of utilization of MWHs at CEmONC facilities [22]. We observed a steep increase in the numbers of women utilizing any MWH for any reason at intervention sites and a slower increase over time at comparison sites (Fig 1, Table 7), which may be attributed to three key drivers. First, the Government of Zambia introduced guidelines in 2018 recommending women deliver at a CEmONC facility if they had: preexisting conditions, prior pregnancy or labor-related complications, a first pregnancy (primigravida), five or more prior pregnancies (multipara), or a multiple gestation [23]. The total utilization increase in time for all sites may reflect better adherence to recommendations regarding risk selection for CEmONC facilities. Second, the CEmONC MWH intervention was happening concurrently with an improved MWH model at BEmONC facilities in the same districts [17]. All CEmONC facilities included in this analysis function as referral sites for BEmONC facilities that may have also received the improved MWH intervention, where waiting women requiring CEmONC care could be identified and referred in a timely manner, in accordance with the national guidelines encouraging risk selection for CEmONC referral. Lastly, the large increase in utilization may be partially related to better record-keeping after the introduction of registers at the MWHs, as the project paid someone to keep records and the project staff offered informal mentorship during the monthly visits to extract the data (and indeed, rates of recording discharge or delivery dates increased during implementation, though less markedly so at comparison sites).

We observed no meaningful differences in the demographic profile of women that used intervention or comparison MWHs, or between the pre- and post-intervention periods with high proportions of primigravida and multipara women, as would be expected under government guidelines. The high degree of missing discharge or delivery data (89%) of women utilizing a comparison MWH limits our ability to interpret the utilization patterns at comparison sites. Though both intervention sites had similar utilization patterns in the pre-intervention period, Zimba clearly drove the spike in post-intervention utilization patterns (Fig 1A–1D). While we observed a relative increase in Nyimba, the difference in Zimba was much more pronounced.

Contextually, Zimba is a mission-run rural hospital in a large very rural district in Southern Province located in a rural area along the main road whereas Nyimba is government-run and located in an urban area along the main road in a relatively small district in Eastern Province (Table 2). Our data do not capture reasons why Zimba experienced such an increase in utilization, but the distinct geographic difference between these sites effectively eliminates concerns of preferential selection by women for one over the other. We hypothesize that the difference in urbanicity between these two intervention sites may result in a differential need for an MWH. A CEmONC facility in a smaller, more urban area may not need a large MWH, whereas MWHs may be essential at more rural CEmONC facilities, such as Zimba, where women travel farther. Such travel distance may also be influencing the high utilization for PNC observed at Zimba. Though there is limited literature on this phenomenon of rural location driving total MWH utilization, it is worth exploring in more depth, particularly if countries are trying to strategically scale MWHs. Also of note, the other mission-run CEmONC facility included in the study, Macha, was a comparison site and did not implement an improved Core MWH Model, though there was also a relative increase of MWH utilization at Macha over the same time period (Fig 1A–1D). While it is possible that there is a perception of improved quality of care at these mission-run CEmONC facilities, the geographic distances between sites likely decreases preferential selection in this setting.

While these data elicit interesting utilization patterns, there are several key limitations. First, intervention sites were purposively selected and are geographically and contextually different from each other as well as from the comparison sites. This limits our ability to compare across sites, rendering this a primarily descriptive analysis. Second, we relied heavily on monitoring data collected from MWH registers, which had missing information, particularly during the pre-intervention time at all sites, and the post-intervention period at comparison sites. Third, this analysis presumes that utilization of an MWH results in care at the CEmONC facility, but we do not report on any health outcomes besides number of deliveries over this time period, which did not vary much between comparison and intervention MWHs. The utilization of an MWH at a CEmONC site in these data does not guarantee that a woman subsequently received care at the CEmONC site or had fewer delivery complications, though a study in Ethiopia suggests that women who use MWHs at hospitals have better birth outcomes than non-MWH users [24,25].

Despite these limitations, to our knowledge, this is the first description of patterns of use at improved MWHs at CEmONC facilities compared to standard of care among rural women in Zambia and corroborates the utilization patterns of MWHs at hospitals in other settings [25]. Our findings stress the importance of appropriate infrastructure as a pre-requisite foundation for a high-quality health system. In these most rural areas of Zambia, CEmONC services cannot be delivered at the rural health clinic level, but MWHs are part of the infrastructure that may help facilitate women being at the correct place for care if needed. In light of the recent Lancet Global Health Commission on High Quality Health Systems, which proposes the paradigm shift of “right place” high quality of care, as opposed to a focus primarily on access to health services at the global level [23], and the Government of Zambia’s referral policies [26] that will inevitably generate demand at CEmONC sites, MWHs at CEmONC sites can play an important role in facilitating compliance for “right place care” at high-volume referral sites. Critically, utilization of the MWH in rural Zimba Mission Hospital for specifically PNC care indicates that MWHs can help ensure that women are able to *remain* at the “right place” for the necessary care for the appropriate length of time.

As the conversation evolves around access to and quality of care for rural populations, the quality and functionality of ‘ancillary’ infrastructure such as MWHs is essential in thinking about how health systems can deliver services to improve maternal and newborn outcomes. Here, we have shown that quality of MWHs at CEmONC facilities can be improved and sustained over time, and that this improved quality may be associated with increased utilization of CEmONC MWHs in rural Zambia. In addition to MWHs potentially providing “right place” care, an important corollary for such infrastructure will be “right size;” as seen with Zimba’s bed occupancy rate greater than 100%, overcrowding at MWHs that are incapable of meeting demand may result in poorer perceived quality. Understanding patterns of CEmONC facility utilization, driven as they may be by ease of accessibility and referral catchment area, may help planning in the future for the “right place" and “right size” of maternal care delivery.

## Supporting information

S1 File MWH utilization register. (PDF)

S2 File Core model checklist for MWH quality assessment. (PDF)

S3 File Focus group discussion guide in English, Tonga, and Nyanja. (PDF)
