# Supplementary material for: Quality and utilization patterns of maternity waiting homes at referral facilities in rural Zambia: A mixed-methods multiple case analysis of intervention and standard of care sites
Source: PLoS One. 2019 Nov 27;14(11):e0225523. doi: 10.1371/journal.pone.0225523 (PMC6881034; doi:10.1371/journal.pone.0225523)
Supplement: S1 File — (PDF) [file pone.0225523.s001.pdf]

Maternity Waiting Home Register
Form D

Instructions for Completing Maternity Waiting Home Register

The Maternity Waiting Home Register will be completed at the Maternity Waiting Home throughout the period of stay of a woman at the Maternity Waiting Home. This register should only be completed with data about pregnant or recent postpartum women (within 8 days of delivery).

Who completes register: The register will be completed by a member of the Maternity Waiting Home management.

Where register will be placed: The Maternity Waiting Home register will be kept at the Maternity Waiting Home

Note: The companion of a women who will soon deliver does not need a separate row of information filled out, just fill out column Q. If an individual who is neither a pregnant woman, postpartum woman, or companion, stays at the Maternity Waiting Home, please register them in the Others Register, not this register. If a woman has left the Maternity Waiting Home to deliver at the health facility, but has returned to the Mothers' Shleter without delivering, write in comments section, do not start a new row of information. If a woman has stayed at the Maternity Waiting Home right before delivery and has returned postpartum within the 8 days after delivery, first complete the delivery date in her previous row of information and begin a new row, completing up to column J.

Description of columns

Column	Column label	Instructions to complete cell
A	MWH regisration number	The MWH registration is a unique number. This is a sequence of letters and number generated as follows:<br>[1] [4 digit province and district code] - [2 digit facility code] - [last 2 digits of the year] - [numbers in ascending order starting at 0001]<br><br>This number should be only given to a woman once. Place a sticker with this number on the woman's ANC card the first time she presents to the MWH. Copy that sticker number into this column each time the woman presents to the Maternity Waiting Home throughout the project.
B	Safe motherhood number	Copy this number from the woman's ANC card in column (a).
C	Date of arrival for this visit	Write the date when the woman reports to the MWH. Format for date: 'dd/mm/yy'
D	Reason for MWH stay for this visit	Write the number of the reason for the person staying at the maternity home, based on the list provided below.<br><br>(1) 1st ANC visit,<br>(2) Other ANC visit,<br>(3) While awaiting delivery<br>(4) After discharge from health facility/ immediately after delivery<br>(5) 3 day post natal visit<br>(6) 7-14 days post natal visit<br>(7) 6 weeks post natal visit<br><br>If the woman does not fit into any of these categories, she does not belong in the MWH register. Please remove her information from the MWH register and complete her information in the Others register
E	Woman's full name	Write the name of the woman in full. First name and then surname (e.g. Mary Daka)
F	Marital Status	Write the womans marital status based on the list provided below.<br><br>(1) Married/Cohabiting<br>(2) Divorced<br>(3) Separated<br>(4) Widowed<br>(5) Never-married
G	Village name	Write the woman's village name
H	Origin code	Write the location of the woman in relation to the institution based on the key provided below<br><br>**HF only:**<br>(1) From within 12km, within the catchment area<br>(2) From more than 12km, within the catchment area<br><br>**Both HF and Hospital:**<br>(3) From within district but outside catchment area.<br>***If 3, please write 3 and the name of the catchment area***<br>(4) From outside district.<br>***If 4, please write 4 and the name of the district***<br>(5) From outside Zambia<br>(97) Unknown

Maternity Waiting Home Register
Form D

I	Date discharged from MWH / Transferred to HF	Write the date when the woman is discharged from the MWH to transferred to the health facility for delivery or for any other reason. Format for date 'dd/mm/yy'
J	Reason for discharge/ transfer	Write the reason of discharge from MWH or transfer to HF based on the key provided below.<br><br>(1) Transferred to HF for delivery or complications<br>(2) Returned home without delivering<br>(3) Returned after ANC visit<br>(4) Returned home after postpartum stay<br>(96) Other. If other, specify
K	Date of delivery	If the woman returns to MWH after delivery write the date when the woman delivered. Format for date 'dd/mm/yy.' Check with health facility stafffor date if woman stayed at MWH before delivery and did not return after delivery.
L	Age at last birthday	Write the age of the woman at her last birthday
M	Highest grade completed	Write the woman's highest level of education completed. Write 00 if no education
N	EDD	Copy this date from the woman's ANC card in column (j). Format for date 'dd/mm/yy'
O	Gravida	Copy this number form the woman's ANC card. If not available, ask the woman" how many times have you been pregnant?" and write the number here
P	Parity	Copy this number form the woman's ANC card. If not available, ask the woman "how many deliveries >20weeks/ 5 months in pregnancy have you had?" and write the number here
Q	Number of previous stillbirths	Ask woman 'how many stillbirths have you had from previous pregnancies?' and write the number here
R	Number of companions — F	Write the number of female companions staying at the MWH with the woman
	Number of companions — M	Write the number of male companions staying at the MWH with the woman
S	Companions stayed at MWH	Provide response for each companion in Column P, separated by a comma.<br><br>(1) Mother<br>(2) Mother in law<br>(3) Aunty<br>(4) Grandmother<br>(5) Sister<br>(6) Child<br>(7) Husband<br>(96) Other. If other, specify
T	Heard of MWH from:	Write the number(s) of how the woman heard about the MWH based on the list provided below. If the woman heard about the MWH from multiple sources, write all numbers, separated by a comma.<br><br>(1) Chief<br>(2) Headman<br>(3) Health care worker<br>(4) SMAG<br>(5) Traditional birth attendant<br>(6) Family member<br>(7) Another mother<br>(8) Other community member<br>(9) Radio<br>(96) Other. If other, specify
U	Transport method used	Write the number of the type of transport that the woman used to come to the MWH based on the list provided below.<br><br>(1) Walking<br>(2) Bicycle<br>(3) Carried in wheelbarrow<br>(4) Animal drawn cart<br>(5) Taxi<br>(6) Car<br>(7) Motorcycle<br>(8) Ambulance<br>(96) Other. If other, specify
V	Travel time from home to Maternity Waiting Home	Write the time it took the woman to travel from home to the MWH, based on the transport method written in column S. Write the number in hours and minutes. If the woman is unable to say how long it took, ask her what time she left home and what time she arrived at the MWH; calculate the length of time.

Maternity Waiting Home Register

Form D

W	Participated in Experience Survey?	If woman participated in the Experience Survey, mark here with 'X'. If she did not participate leave blank
X	Comments	Write any other comments about individuals stay at the MWH here.

District:_______________________

Province: _____________________

Maternity Waiting Home Register
Form D

Health Facility ID: ____________________

A	B	C	D	E	F	G	H	I	J	K
**MWH Registration number**<br>Write the MS registration number here and place a sticker with the same number on the woman's ANC card	**Safe Motherhood Number**<br>(Copy from ANC card)<br>*Write "unavailable" if SMN unavailable*	**Date of arrival to MWH for this visit**<br>(dd/mm/yy)	**Reason for MWH stay for this visit**<br>(1) 1st ANC visit,<br>(2) Other ANC visit,<br>(3) While awaiting delivery<br>(4) After discharge from health facility/ immediately after delivery<br>(5) 3 day post natal visit<br>(6) 7-14 days post natal visit<br>(7) 6 weeks post natal visit	**Woman's full name**<br>(First name, Surname)	**Marital Status**<br>(1) Married/Cohabiting<br>(2) Divorced<br>(3) Separated<br>(4) Widowed<br>(5) Never-married	**Village name**	**Origin code**<br>**HF only:**<br>(1) From within 12km, within the catchment area<br>(2) From more than 12km, within the catchment area<br>**Both HC and Hospital:**<br>(3) From within district but outside catchment area<br>(4) From outside district<br>(5) From outside Zambia<br>(97) Unknown	**Date discharged from MWH/ Transferred to HF**<br>(dd/mm/yy)	**Reason for Discharge / Transfer**<br>(1) Transferred to HF for delivery or complications,<br>(2) Returned home without delivering,<br>(3) Returned home after ANC visit<br>(4) Returned home after postpartum stay<br>(96) Other. If other, specify	**Date of delivery**<br>If returning from facility after delivery<br>(dd/mm/yy)<br>***If woman did not return to the MWH after delivery, check with health facility staff for delivery date and enter here**
_ - _ _ _ _ - _ _ _ - _ _ _ - _ _ _ _ _ _	_ _ _ _ - _ _ _ - _ _ _ _ _ - _ _ _	/ /						/ /		/ /
_ - _ _ _ _ - _ _ _ - _ _ _ - _ _ _ _ _ _	_ _ _ _ - _ _ _ - _ _ _ _ _ - _ _ _	/ /						/ /		/ /
_ - _ _ _ _ - _ _ _ - _ _ _ - _ _ _ _ _ _	_ _ _ _ - _ _ _ - _ _ _ _ _ - _ _ _	/ /						/ /		/ /
_ - _ _ _ _ - _ _ _ - _ _ _ - _ _ _ _ _ _	_ _ _ _ - _ _ _ - _ _ _ _ _ - _ _ _	/ /						/ /		/ /
_ - _ _ _ _ - _ _ _ - _ _ _ - _ _ _ _ _ _	_ _ _ _ - _ _ _ - _ _ _ _ _ - _ _ _	/ /						/ /		/ /
_ - _ _ _ _ - _ _ _ - _ _ _ - _ _ _ _ _ _	_ _ _ _ - _ _ _ - _ _ _ _ _ - _ _ _	/ /						/ /		/ /
_ - _ _ _ _ - _ _ _ - _ _ _ - _ _ _ _ _ _	_ _ _ _ - _ _ _ - _ _ _ _ _ - _ _ _	/ /						/ /		/ /
_ - _ _ _ _ - _ _ _ - _ _ _ - _ _ _ _ _ _	_ _ _ _ - _ _ _ - _ _ _ _ _ - _ _ _	/ /						/ /		/ /
_ - _ _ _ _ - _ _ _ - _ _ _ - _ _ _ _ _ _	_ _ _ _ - _ _ _ - _ _ _ _ _ - _ _ _	/ /						/ /		/ /
_ - _ _ _ _ - _ _ _ - _ _ _ - _ _ _ _ _ _	_ _ _ _ - _ _ _ - _ _ _ _ _ - _ _ _	/ /						/ /		/ /
_ - _ _ _ _ - _ _ _ - _ _ _ - _ _ _ _ _ _	_ _ _ _ - _ _ _ - _ _ _ _ _ - _ _ _	/ /						/ /		/ /
_ - _ _ _ _ - _ _ _ - _ _ _ - _ _ _ _ _ _	_ _ _ _ - _ _ _ - _ _ _ _ _ - _ _ _	/ /						/ /		/ /

District:________________________

Province: ______________________

Maternity Waiting Home Register
Form D

Health Facility ID: ______________________

L	M	N	O	P	Q	R		S	T	U	V		W	X
**Age at last birthday**	**Highest grade completed**<br>Write 00 for no education	**EDD**<br>(dd/mm/yy)	**Gravida**<br>Copy from ANC card if available<br>If not available, ask the woman 'how many times have you been pregnant?' and write the number here	**Parity**<br>Copy from ANC Card if available<br>If not available, ask the woman 'how many deliveries >20weeks/ 5 months in pregnancy have you had?' and write the number here	**Number of previous still births**<br>Ask woman 'how many stillbirths have you had from previous pregnancies?' and write the number here	**Number of companions**		**Companions**<br>Provide response for each companion:<br>(1) Mother<br>(2) Mother-i- law<br>(3) Aunty<br>(4) Grandmother,<br>(5) Sister<br>(6) Child<br>(7) Husband<br>(96) Other.<br>If other, specify	**Heard of MS from:**<br>*(select all that apply)*<br>(1) Chief<br>(2) Headman<br>(3) Health care worker<br>(4) SMAG<br>(5) Traditional birth attendant<br>(6) Family member<br>(7) Another mother<br>(8) Other community member<br>(9) Radio<br>(96) Other<br>If other, specify.	**Transport method used**<br>(1) Walking<br>(2) Bicycle<br>(3) Carried in wheelbarrow<br>(4) Animal drawn cart<br>(5) Taxi<br>(6) Car<br>(7) Motorcycle<br>(8) Ambulance<br>(96) Other<br>If other, specify.	**Travel time from home to mothers' shelter**		**Participated in Experience Survey?**<br>Tick here if participated in experience survey	**Comments**
						**F**	**M**				**Hr**	**Min**		
__ __		/ /												
__ __		/ /												
__ __		/ /												
__ __		/ /												
__ __		/ /												
__ __		/ /												
__ __		/ /												
__ __		/ /												
__ __		/ /												
__ __		/ /												
__ __		/ /												
__ __		/ /												
