# Supplementary material for: Quality and utilization patterns of maternity waiting homes at referral facilities in rural Zambia: A mixed-methods multiple case analysis of intervention and standard of care sites
Source: PLoS One. 2019 Nov 27;14(11):e0225523. doi: 10.1371/journal.pone.0225523 (PMC6881034; doi:10.1371/journal.pone.0225523)
Supplement: S2 File — (PDF) [file pone.0225523.s002.pdf]

Today's Month and Year (MM/YYYY): ___ ___ /___ ___ ___ ___
Facility ID: ___________

Core Model Checklist

Maternity Homes Alliance

Instructions: This assessment should be performed ***monthly*** at the Maternity Waiting Home (MWH) by project staff through observations and conversations with MWH and health facility staff. The data calendar month and year should be for the day you observe the MWH. Tick the correct response based on your observations and discussions. Provide any further comments when appropriate.

Province: ___________________________

District: ____________________________

Health Facility Name: ___________________________________

Health Facility ID: ☐☐☐☐☐☐

Date of interview: ☐☐ ☐☐ ☐☐☐☐

D D M M Y Y Y Y

Data Collector Name: ________________

Section I:

Question	Response	Comments
SS1. Does this site have a designated space for waiting mothers?<br><br>*Probe: ask if pregnant mothers come to wait at the health facility before delivering and then where do they wait.*	○ Yes (1)<br>○ No (0)	If (0), do not proceed but please elaborate here:
SS2. If yes, what is this space:	○ A MAHMAZ Maternity Waiting Home (1)<br>○ An existing shelter (2)<br>○ A relatives structure (3)<br>○ An alternative structure (4)<br>○ A ward (5)<br>○ Other (6) (specify):	
SS3. Do women currently use this space as they wait for delivery?	○ Yes (1)<br>○ No (0)	
Instructions: *Ask to see the MWH register. Find out when the last woman reported to the MWH then*		

Today's Month and Year (MM/YYYY): ___ ___ /___ ___ ___ ___
Facility ID: ___________

check and see if she was entered into the MWH register	
Was the last woman who reported to the MWH registered into the register?	○ Yes (1)<br>○ No (0)

Section II:

Question	Response	Comment
1. Today's Date (dd/mm/yyyy)	____/ ______/_______<br>*(dd) (mm) (yyyy)*	
**Infrastructure, Equipment, & Supplies**		
Observe the shelter's structure and interior, its walls, roof, and floor		
2. Are the walls intact (no major cracks)?	○ Yes (1)<br>○ No (0)<br>○ N/A (96)	
2a. What are the walls made of?<br><br>*If more than one material, select the one that is "most" common.*	*NATURAL WALLS*<br>NO WALLS (0)<br>CANE/PALM/TRUNKS (1)<br>MUD (2)<br>*RUDIMENTARY WALLS*<br>BAMBOO/POLE WITH MUD (3)<br>STONE WITH MUD (4)<br>PLYWOOD (5)<br>CARDBOARD (6)<br>REUSED WOOD (7)<br>*FINISHED WALLS*<br>CEMENT (8)<br>STONE WITH LIME/CEMENT (9)<br>BRICK (10)<br>CEMENT BLOCKS (11)<br>WOOD PLANKS (12)<br>OTHER (SPECIFY) (13):	
3. Does the roof have any noticeable holes?	○ Yes (1)<br>○ No (0)<br>○ N/A (96)	If (96), Skip to 5
4. Does the roof show any signs of leaking?	○ Yes (1)<br>○ No (0)	
4a. What is the roof made of?<br>*If more than one material, select the one that is "most" common.*	*NATURAL ROOFING*<br>NO ROOF (0)<br>THATCH/PALM LEAF (1)<br>*RUDIMENTARY ROOFING*<br>RUSTIC MAT (2)<br>PALM/BAMBOO (3)<br>WOOD PLANKS (4)<br>CARDBOARD (5)<br>*FINISHED ROOFING*<br>METAL/IRON SHEETS (6)<br>WOOD (7)<br>CALAMINE/CEMENT FIBRE (ASBESTOS) (8)<br>CERAMIC/HARVEY TILES (9)	

Today's Month and Year (MM/YYYY): ___ ___ /___ ___ ___ ___
Facility ID: ___________

	CEMENT (10)<br>ROOFING SHINGLES (11)<br>MUD TILES (12)<br>OTHER (SPECIFY) (13):	
5. Are the floors intact (no major cracks)?	o Yes (1)<br>o No (0)<br>o N/A (96)	
5a. What are the floors made of?<br><br>*If more than one material, select the one that is "most" common.*	<u>NATURAL FLOOR</u><br>EARTH/SAND (1)<br>DUNG (2)<br><u>RUDIMENTARY FLOOR</u><br>WOOD PLANKS (3)<br>PALM/BAMBOO/REEDS (4)<br><u>FINISHED FLOOR</u><br>PARQUET/POLISHED WOOD (5)<br>VINYL (PVC) OR ASPHALT STRIPS (6)<br>CERAMIC/TERRAZZO TILES (7)<br>CONCRETE CEMENT (8)<br>CARPET (9)<br>OTHER (SPECIFY) (10):	
6. In general, how clean is the shelter?	o Sufficiently clean (1)<br>o Clean but needs improvement (2)<br>o Not clean (3)	
Comments:		
Observe the doors and windows in the shelter. Open and close the doors and windows. Lock and unlock the doors and windows.		
7. Do all doors used to enter the shelter open and close?	o Yes (1)<br>o No (0)<br>o N/A (96)	If (96), Skip to 9
8. Do all doors used to enter the shelter lock?	o Yes (1)<br>o No (0)	
9. Are all windows intact (in frame and panes unbroken)?	o Yes (1)<br>o No (0)<br>o N/A (96)	If (96), Skip to 12
9a. Do all windows have screens?	o All<br>o Most<br>o Some<br>o None	
10. Do all windows open and close?	o Yes (1)<br>o No (0)	
11. Do all windows lock?	o Yes (1)<br>o No (0)	
Comments:		
Observe the lighting within the shelter. Turn the light source on and off.		

Today's Month and Year (MM/YYYY): ___ ___ /___ ___ ___ ___
Facility ID: ___________

12. Did the light turn on?	○ Yes (1)<br>○ No (0)<br>○ N/A (96)	If (96) or (0), Skip to 14
13. Is there enough lighting to light two rooms at once?	○ Yes (1)<br>○ No (0)	
Comments:		
Observe the beds, bedding, and bed nets		
**Question**	**Response**	**Comment**
14. How many bed frames are there in the shelter (total broken and unbroken)?<br>a. Hospital grade (waist high)?<br>b. Non-hospital grade?	a.________<br>b.________	If none, Skip to 17a
15. How many bed frames are broken?<br>a. Hospital grade (waist high)<br>b. Non-hospital grade?	a.________<br>b.________	
16. Does each frame have a mattress on it?	○ All (1)<br>○ Most (2)<br>○ Some (3)<br>○ None (4)	
17. Does each bed have a mattress under it or nearby?	○ All (1)<br>○ Most (2)<br>○ Some (3)<br>○ None (4)	If (4) for both 16 and 17, skip to 20<br><br>If (1), (2), or (3), skip to 18
17a. If there are no bed frames, how many mattresses are on the floor (that are owned by the clinic)?<br>*If none, write 00.*	|___|___|	If none, skip to 20
18. Are the mattresses covered in plastic?	○ All (1)<br>○ Most (2)<br>○ Some (3)<br>○ None (4)	
19. Are clean sheets available for each mattress?	○ All (1)<br>○ Most (2)<br>○ Some (3)<br>○ None (4)	
20. Are mosquito nets over each mattress?	○ All (1)<br>○ Most (2)<br>○ Some (3)<br>○ None (4)	If (4), Skip to 22
21. Are mosquito nets intact (have no holes)?	○ All (1)<br>○ Most (2)<br>○ Some (3)<br>○ None (4)	
Comments:		

Today's Month and Year (MM/YYYY): ___ ___ /___ ___ ___ ___
Facility ID: ___________

Observe the presence of cabinets and the storage of food		
22. Are there lockable cupboards accessible to women staying at the shelter?	○ Yes (1)<br>○ No (0)	If (0), skip to 25
23. Is the lock available for each lockable cupboard?	○ All (1)<br>○ Most (2)<br>○ Some (3)<br>○ None (4)	If (4), skip to 25
24. Are keys available for all locks on cupboards?	○ All (1)<br>○ Most (2)<br>○ Some (3)<br>○ None (4)	
25. Is food stored properly (e.g. closed containers, away from pests and water)?	○ Yes (1)<br>○ No (0)	
Comments:		
Observe the space within the shelter for postnatal women and newborns to stay.		
26. Is there a separate space within the shelter for postnatal women and newborns to stay?	○ Yes (1)<br>○ No (0)	
Comments:		
Observe the staff room. Staff room must be specifically for the Maternity Waiting Home and not just a general health facility room.		
27. Is there a functional desk and chair?	○ Yes (1)<br>○ No (0)<br>○ No staff room (2)	If (2), Skip to 24
28. Is there a functioning lockable cabinet for records?	○ Yes (1)<br>○ No (0)	
Comments:		
Observe the water source.		
29. Is potable water available and accessible?	○ Yes (1)<br>○ No (0)	If (0), Skip to 31
30. *Draw water from the source.* Can you draw water?	○ Yes (1)<br>○ No (0)	
Comments:		
Observe the cooking area and supplies.		

Today's Month and Year (MM/YYYY): ___ ___ /___ ___ ___ ___
Facility ID: ___________

31. Is there a designated space for cooking?	○ Yes (1)<br>○ No (0)	If (0), skip to 33
31a. Is the cooking area large enough for 5 women to cook in at one time?	○ Yes (1)<br>○ No (0)	
31b. In general, is the cooking area being used by the women?<br>*Ask the manager or a woman*	○ Yes (1)<br>○ No (0)	If (1), skip to 32
31c. If no, why not?		
32. Is the cooking area covered?	○ Yes (1)<br>○ No (0)	
33. Are there functional cooking pots and utensils available?	○ Yes (1)<br>○ No (0)	
Comments:		
Observe the bathing and laundry areas.		
34. Do mothers have access to a private bathing area?	○ Yes, a separate area (1)<br>○ Yes, but bathing from the latrines (2)<br>○ No (0)<br>○ N/A (96)	If (0) or (96), Skip to 36
35. In general, how clean is the bathing area?	○ Sufficiently clean (1)<br>○ Clean but needs improvement (2)<br>○ Not clean (3)	
36. Is there a private area where women can dry their clothes after washing?	○ Yes (1)<br>○ No (0)<br>○ N/A (96)	
Comments:		
Observe the latrines.		
37. Does the latrine have ventilation?	○ Yes (1)<br>○ No (0)<br>○ N/A (96)	If (96), Skip to 40
38. In general, how clean is the latrine?	○ Sufficiently clean (1)<br>○ Clean but needs improvement (2)<br>○ Not clean (3)	
39. Is the latrine functional today?	○ Yes (1)<br>○ No (0)	

Today's Month and Year (MM/YYYY): ___ ___ /___ ___ ___ ___
Facility ID: ___________

Comments:		
Observe the grounds around the shelter.		
40. In general, are the grounds around the shelter clean?	○ Sufficiently clean (1)<br>○ Clean but needs improvement (2)<br>○ Not clean (3)	
Comments:		
Observe the IGA activities (tailoring and keyhole garden) – ONLY for Intervention Sites, skip if Control Site		
41a. Are there any tailors working at this facility?	○ Yes (1)<br>○ No (0)	If (0), skip to 41c
Comments:		
41b. What kind of products are being made?<br><br>*Select all that apply.*	○ Sanitary pads (1)<br>○ Baby clothes (including hats, shawls, socks, etc) (2)<br>○ Blankets and quilts (3)<br>○ School uniforms(4)<br>○ Fabric nappies (5)<br>○ Mealie meal carryon bags (6)<br>○ Other (7) Please specify__________	
41c. How many sewing machines are there total?<br>*If none, write 00.*	|___|___|	
41d. How many sewing machines are currently working? How many are broken?<br><br>*If none, write 00.*	|___|___| Working<br><br>|___|___| Broken	
41e. Is there a garden for this MWH?	○ Yes (1)<br>○ No (0)	If (0), skip to 41
41f. What type of garden is it?	○ Keyhole (1)<br>○ Other (2) Please specify__________<br>○ Both (3)	

41g. Has the garden produced food in the last month?	○ Yes (1)<br>○ No (0)	If (1), skip to 41
41h. If no, why not?<br>*Ask the manager.*		
**Section III: Policies, Management, & Finances**		
Ask to see the shelter's mission statement, standard operating procedures, and accounting books.		
42. Does this shelter have a governance committee?	○ Yes, a committee specific for the MWH (1)<br>○ Yes, the HF committee governs the MWH as well (2)<br>○ No (0)	
Comments:		
43. Does the shelter have a manager?<br><br>*There is a person that can be identified as a shelter manager (either paid or volunteer)*	○ Yes (1)<br>○ No (0)<br>○ No position exists (2)	If (0) or (2), skip to 44
Comments:		
44. Does the manager believe that a job description(s) for managing the shelter exists?	○ Yes (1)<br>○ No (0)	
45. Is the mission statement easily accessible/visible?<br><br>*Verify by sight*	○ Yes (1)<br>○ No (0)<br>○ N/A (96)	
46. Are the standard operating procedures written and available to observe at time of assessment?	○ Yes (1)<br>○ No (0)<br>○ N/A (96)	
47. Are the accounting books up to date?	○ Yes (1)<br>○ No (0)<br>○ N/A (96)	If (0), skip to 49
48. Are standard accounting procedures being used?<br>*Ask to see the financial management manual (it should be easily accessible), which has the standard accounting procedures defined in there.*	○ Yes (1)<br>○ No (0)<br>○ Don't know (97)	

49. Is there a process/mechanism in place for women to give feedback on their shelter experiences that is separate from the study experience survey?	○ Yes (1)<br>○ No (0)<br>○ Don't know (97)	
50. Please tell me any methods that are in place to learn about your opinions of the shelter.<br><br>*Select all that apply.*	○ Suggestion box (1)<br>○ Client survey form (separate from this one) (2)<br>○ Official meeting with community leaders (3)<br>○ Informal discussion with community (4)<br>○ Letters (5)<br>○ Other (6) (specify):___________	
Comments:		
**Section IV: Linkages with Health Facility**		
Observe the location of the shelter in relation to the health facility (BEmONC). Ask the manager or HF representative about the location of a CEmONC-capable facility (i.e. a referral hospital) and the emergency transport system.		
51. Is the shelter nearby the health facility?	○ Yes (1)<br>○ No (0)	
52. If needed, could a woman get to CEmONC within 2 hours?	○ Yes (1)<br>○ No (0)<br>○ NA/CEmONC (2)	*Why/why not?*
53. Does the health facility have a transport system identified for referrals?	○ Yes (1)<br>○ No (0)<br>○ NA/CEmONC (2)	If (0) or (2), skip to 54
54. What is the mode of referral transportation and availability?		

END OF SURVEY
