# Supplementary material for: Quality and utilization patterns of maternity waiting homes at referral facilities in rural Zambia: A mixed-methods multiple case analysis of intervention and standard of care sites
Source: PLoS One. 2019 Nov 27;14(11):e0225523. doi: 10.1371/journal.pone.0225523 (PMC6881034; doi:10.1371/journal.pone.0225523)
Supplement: S3 File — (PDF) [file pone.0225523.s003.pdf]

GROUP ID

Instrument ID: Form K5

The MAHMAZ Project

Focus Group Discussion Guide- ENGLISH

Target Audience:

1. *Pregnant or recently delivered (in the past 30 days) women who have been at CEmONC MS for at least 1 night*
2. *Women >=15 years at last birthday*
3. *Has not previously participated in a FGD in the same year at another site or in another category*

Short screen:

Criteria	Response	Comments
SS1. Is the woman currently pregnant or recently delivered (in the past 30 days) and has stayed at least 1 night at the MWH?	Yes (1)<br>No (0)	If (0), do not proceed. Woman is not eligible.
SS2. Is the woman >=15 years old?	Yes (1)<br>No (0)	If (0), do not proceed. Woman is not eligible.
SS3. Has the woman previously participated in a focus group in the same year at the same or another site or other categories?	Yes (1)<br>No (0)	If (1), do not proceed. Woman is not eligible.

Instructions for the interviewer

Step 1: Informed consent: Ask each participant for a few minutes of their time. Introduce yourself and the study. Begin the informed consent process as per the training.

Was informed consent obtained and documented for each participant before the start of the FGD?

YES ______ (proceed with roster and FGD)

NO ______ (STOP! Thank the participant for their time but ask any who did not agree to leave. Then proceed with the FGD.)

INSTRUMENT K5: GROUP ID

Focus Group Discussion Guide for CEmONC Maternity Waiting Home

Interviewer: *Read the following statement. Please repeat the statement translated into the local language based on primary languages used by the group.*

"Thank you for agreeing to participate in this interview. My name is _____. I will be asking you the questions. My partner _____ will be taking notes on the things you have to say. We will record this session.

We want to understand your views on Maternity Waiting Homes and access to maternity care at the health facility in your community. Please feel free to tell us whatever you are comfortable sharing. As a reminder, please do not share anything you hear or said within this group outside of this group. You should also remember that you do not have to share anything you are not comfortable sharing. We will not link your information to your responses. There are no right or wrong answers, so please be honest and us what is true for you and your community. Are you ready to begin?"

Step 2: *Please complete the roster table for each participant as they sign in. Verify eligibility. This form will have an ID letter for each participant. Make sure that the note taker has the correct ID letter recorded on his/her notes prior to beginning and that participants have their correct "Letter Label".*

Step 3: *Proceed to the FGD. Start by reiterating the importance of confidentiality within the group. As you ask the questions, please probe to obtain as much information as possible for each question.*

Facilitator name ___________________ **Note-taker Name ___________________**

1. **FGD Date (DD/MM/YYY) ________________**
2. **Health Facility Name _________________**
3. **Health Facility ID _________________**
4. **District Name _________________**
5. **Province Name _________________**
6. **Time start (HH:MM) ________** **Time finish (HH:MM) ________**
7. **Supervisor initials __________**

INSTRUMENT K5: GROUP ID

Focus Group Discussion Guide for CEmONC Maternity Waiting Home

Demographics: Pregnant/ Recently Delivered Women

ID	Age at last birthday	Woman is pregnant?<br>1/0	How many months is the pregnancy?	Woman recently delivered?<br>1/0	How old is baby?	Prev FGD? 1/0	Eligibility confirmed? 1/0<br>If 1, proceed with the rest of the columns<br>If 0, stop and thank the woman for her time	Gender<br>Male (1)<br>Female (2)	Marital status:<br>Married/ cohabiting (1)<br>Divorced (2)<br>Separated (3)<br>Widowed (4)<br>Never married (5)	What is the highest grade you completed?<br>If <1, put 0.<br>If >12, put 13.	How many children do you have?	Total # of pregnancies including current	For your most recent pregnancy, where did/will you deliver?<br>In a home (1)<br>Health facility (2)<br>Hospital (3)<br>Other (4) (specify)	What HF are you closest to?	Travel time to this MWH (include units – minutes or hours)
**A**								**2**							
**B**								**2**							
**C**								**2**							
**D**								**2**							

**E**								**2**							
**F**								**2**							
**G**								**2**							
**H**								**2**							

Theme 1: Awareness, Utilization and Quality of the Maternity Waiting Homes

1a. Tell me what you know ***about Maternity Waiting Homes in general.***

i. What are they for?
ii. Who can stay there?

1b. What have you observed about the ***quality of the Maternity Waiting Home*** at this hospital?

Probe for:

- Comfort (Structure, furnishings, bedding, bed nets, cultural appropriateness)
  - Crowding
- Safety (lockable doors & windows, lockable cabinets for belongings)
- Cleanliness
- Food availability
- Seasonal problems (leaking roofs, insect problems, etc)

1c. Did you have to share a bed or mattress during stay?
If yes, please explain why

1d. What have you observed about the spaces for food storage and cooking?
- If no cooking space, where do women cook and store food?

1e. What have you observed about the ***management of the Maternity Waiting Home***?

i. Is there either a community member or health facility staff who helps with the operation of this Maternity Waiting Home? Does anyone seem to be 'in charge' who you met with when you came to stay at the Maternity Waiting Home?

1f. If there are ***maintenance concerns about the MWH***, do you know where to go to get this addressed?

1g. Who would you go to if there was a ***problem at the MWH***?

1h. What have you observed about the ***linkages between the Maternity Waiting Home and the hospital***?

i. During your stay have hospital staff come to shelter to check on women?
ii. Do waiting women attend ANC check-ups at the hospital? How often?

1i. What things make it ***hard for a woman to use the Maternity Waiting Home***?
Probe for: (ask about each bullet separately, do not ask all at once!)

- Quality of Maternity Waiting Home (safety, comfort, cultural appropriateness, other problems)
- Costs
- Food or cooking space
- Perceived quality of care at the health facility
- Family member opinions
- Other responsibilities at home
- Season

1j. What things make it ***easy for a woman to use the Maternity Waiting Home***?
Probe for: (ask about each bullet separately, do not ask all at once!)

- Quality of Maternity Waiting Home (safety, comfort, cultural appropriateness, other good things)
- Costs
- Food or cooking space
- Perceived quality of care at the health facility
- Family member opinions
- Other responsibilities at home
- Season

1k. What are some of the ***reasons you chose to come*** to wait at a Maternity Waiting Home at this hospital?

i. Were you referred from **ANC** to come to this hospital?
ii. Were you referred from **PNC** to come to this hospital?
iii. Is the closest health facility for them?
iv. Was your perception of the MS a factor?
v. How did you know about this Maternity Waiting Home?

1l. Was this this ***first MS*** you have stayed at for this pregnancy?

- If no, why did you move?

Theme 2: Community Ownership and Sustainability of Maternity Waiting Homes

2a. How does the ***community contribute towards maintenance/upkeep*** of the Maternity Waiting Homes or structure at your health facility?

Probe for:

- Cash contributions
- Food contributions
- In-kind labor (slashing, weeding, sweeping, etc)
- Maintenance
- Any other contributions?

2b. Did you have to ***pay or contribute*** anything in kind to stay at this Maternity Waiting Home? Explain.

Theme 3: Barriers and facilitators to facility delivery

3a. Why did you choose to ***deliver at this hospital instead of at home***?

Probe for:

- Being referred by ANC
- Distance/transport (ask for specifics)
- Costs (ask for specifics)
- Seasons (ask for specifics)
- Household responsibilities
- Permission or support from family members
- Perceptions of health facility and quality of care
- Perceptions of pregnant women's or child's health risk
- Myths and misconceptions about home delivery

3b. Why did you choose to ***deliver at this hospital instead of at rural health facility***?

Probe for:

- Being referred by ANC
- Distance/transport (ask for specifics)
- Costs (ask for specifics)
- Seasons (ask for specifics)
- Household responsibilities
- Permission or support from family members
- Perceptions of health facility and quality of care
- Perceptions of pregnant women's or child's health risk
- Myths and misconceptions about home delivery

3c. In your opinion, does a Maternity Waiting Home ***influence a woman/family's decision*** to deliver at a hospital? Why or why not?

<u>Theme 4: Preparedness and Costs</u>

4a. What ***costs*** do women have when ***delivering at this hospital***?

Probe for:

- Birth supplies including plastic sheets, gloves, sterile blade, etc
- Baby clothes
- Travel
- Food
- Payments for delivery, informal payments, in-kind contributions to birth attendant

4b. How do these costs differ when a woman ***delivers at home***?

Probe for:

- Birth supplies including plastic sheets, gloves, sterile blade, etc
- Baby clothes
- Travel
- Food
- Payments for delivery, informal payments, in-kind contributions to birth attendant

4c. How do these costs differ when a woman ***delivers at a rural health centre***?

Probe for:

- Birth supplies including plastic sheets, gloves, sterile blade, etc
- Baby clothes
- Travel
- Food
- Payments for delivery, informal payments, in-kind contributions to birth attendant

4d. How much do women normally ***spend on birth supplies***? Where do women purchased them?

Probe for:

- Plastic sheet, gloves, sterile blade/razor, clamps
- Anything else?

4f. Where do women normally ***purchase baby clothes***?

4h. What ***modes of transport*** do women use to get to the hospital for delivery?

4k. How much do women normally ***spend on food*** for during their stay at the MS? (Separate from what they share with home)

4l. Did you ***save any money*** for the costs of traveling to the hospital and in preparation for delivery here? How did you save?

4m. What are the main ***challenges associated with saving money*** for delivery?
Probe with:

- Competing needs (accidents, illness, basic needs)
- Safety of storing money

This is the end of the Focus Group. Please thank the participants for their time.

GROUP ID

Instrument ID: Form K5

The MAHMAZ Project

Focus Group Discussion Guide - ENGLISH/TONGA

Target Audience:

1. *Pregnant or recently delivered (in the past 30 days) women who have been at CEmONC MWH for at least 1 night*
2. *Women >=15 years at last birthday*
3. *Has not previously participated in a FGD in the same year at another site or in another category*

Short screen:

Criteria	Response	Comments
SS1. Is the woman currently pregnant or recently delivered (in the past 30 days) and has stayed at least 1 night at the MS?	Yes (1)<br>No (0)	If (0), do not proceed. Woman is not eligible.
SS2. Is the woman >=15 years old?	Yes (1)<br>No (0)	If (0), do not proceed. Woman is not eligible.
SS3. Has the woman previously participated in a focus group in the same year at the same or another site or other categories?	Yes (1)<br>No (0)	If (1), do not proceed. Woman is not eligible.

Instructions for the interviewer

Step 1: Informed consent: Ask each participant for a few minutes of their time. Introduce yourself and the study. Begin the informed consent process as per the training.

Was informed consent obtained and documented for each participant before the start of the FGD?

YES ______ **(proceed with roster and FGD)**

NO ______ **(STOP! Thank the participant for their time but ask any who did not agree to leave. Then proceed with the FGD.)**

Interviewer: *Read the following statement. Please repeat the statement translated into the local language based on primary languages used by the group.*

"Twalumba kuti mwazumina kutola lubazu mukuvwuntauzya ooku. Mazina angu idime _____. Ndime ndinikumibuya mibuzyo. Oooyu godililimwi ngu _____ walo ngunikulemba zyonse zyomulaambe, alimwi tulabelesya kancini kulekoda zyotunikubandika.

Tuyanda kuzyiba zyomuyeya ang'anda yabamatumbu ambomujana nseba na bubambe bujatikizya kutumbuka muno mucooko cenu. Ndakomba amwaanguluke kutwaambila zyomuyeya kuti ngamwatwaambila. Tulamuyezya kuti zyonse zyomulabandike anon a kuvwa aano, mutakazyaambi kunze awawa. Alimwimwezele kuyeya kuti na kuli cintu ncomutalimvwi kabotu kwaamba ano, mutacaambi. Tatukwe tugumikizyane zyomulaambe kuli zyomula vwuwe. Kunyina ziluzi azitaluzi pe, Twalomba kuti mwambe zyakasimpe zigaminina ndunwe a zyamucooko cenu. Sena mwalibambila kuti tutalike?"

Step 2: *Please complete the roster table for each participant as they sign in. Verify eligibility. This form will have an ID letter for each participant. Make sure that the note taker has the correct ID letter recorded on his/her notes prior to beginning and that participants have their correct "Letter Label".*

Step 3: *Proceed to the FGD. Start by reiterating the importance of confidentiality within the group. As you ask the questions, please probe to obtain as much information as possible for each question.*

Facilitator name ____________________ **Note-taker Name ____________________**

1. **FGD Date (DD/MM/YYY) ________________**
2. **Health Facility Name __________________**
3. **Health Facility ID __________________**
4. **District Name __________________**
5. **Province Name __________________**
6. **Time start (HH:MM) ________** **Time finish (HH:MM) ________**
7. **Supervisor initials __________**

INSTRUMENT K5:
Focus Group Discussion Guide for CEmONC Maternity Waiting Homes

GROUP ID

Demographics: Pregnant/ Recently Delivered Women

ID	Age at last birthday	Pregnant or recently delivered<br><br>Pregnant (1)<br><br>Recently delivered (2)	If pregnant, how many months is the pregnancy?	If recently delivered, age of baby months (if <1 month, put 0)	Prev FGD this Round?<br><br>Yes (1)<br>No (0)<br><br>If 1, person not eligible to participate	Eligibility confirmed? 1/0<br><br>If 1, proceed with the rest of the columns<br><br>If 0, stop and thank the woman for her time	Gender<br>Male (1)<br>Female (2)	Marital status:<br><br>Married/ cohabiting (1)<br><br>Divorced (2)<br><br>Separated (3)<br><br>Widowed (4)<br><br>Never married (5)	What is the highest grade you completed?<br><br>If < grade 1, put 0. If > grade 12, put 13.	How many live, biological children do you have?	Total # of pregnancies including current one	For your most recent pregnancy, where did/will you deliver?<br>In a home (1)<br>Health facility (2)<br>Hospital (3)<br>Other (4)<br>(specify)	What HF are you closest to?	Travel time to this MS (include units – minutes or hours)
**A**							**2**							
**B**							**2**							
**C**							**2**							
**D**							**2**							

INSTRUMENT K5:
Focus Group Discussion Guide for CEmONC Maternity Waiting Homes

GROUP ID

ID	Age at last birthday	Pregnant or recently delivered<br>Pregnant (1)<br>Recently delivered (2)	If pregnant, how many months is the pregnancy?	If recently delivered, age of baby months (if <1 month, put 0)	Prev FGD this Round?<br>Yes (1)<br>No (0)<br>If 1, person not eligible to participate	Eligibility confirmed? 1/0<br>If 1, proceed with the rest of the columns<br>If 0, stop and thank the woman for her time	Gender<br>Male (1)<br>Female (2)	Marital status:<br>Married/ cohabiting (1)<br>Divorced (2)<br>Separated (3)<br>Widowed (4)<br>Never married (5)	What is the highest grade you completed?<br>If < grade 1, put 0. If > grade 12, put 13.	How many live, biological children do you have?	Total # of pregnancies including current one	For your most recent pregnancy, where did/will you deliver?<br>In a home (1)<br>Health facility (2)<br>Hospital (3)<br>Other (4) (specify)	What HF are you closest to?	Travel time to this MS (include units – minutes or hours)
**E**							**2**							
**F**							**2**							
**G**							**2**							
**H**							**2**							

Theme 1: Awareness, Utilization and Quality of the MWH

1a. Kamu twaambila ncomuzyi ang'anda yaba matumbu/ yaba mamama?

Tell me what you know ***about MWHs in general.***

i. Ila mumlimo nzi? (What are they for?)
ii. Mbani belede kukala mumo? (Who can stay there?)

1b. Ciinzi nco mwabona abube bwa ang'anda yabama kaintu na mayake a cibadela eci?

What have you observed about the ***quality of the MWH*** at this hospital?

Probe for:

- Kukala kabotu? (mayake, zijanika mukati mwa ng'anda, zibelesho, zyimwi zyintu zyijatikizya kuona kabotu, kuyanzana/kujatana kabotu akukala kwa bantu/tunsiya-nsiya).
  Comfort (Structure, furnishings, bedding, bed nets, cultural appropriateness)
- Kwaata (Crowding)
- Kukala kunyina kuyowa (Kukomeka kwa zyijazyo, ma window amomubika zyimwi zyintu zyenu mbuli ma cabati
  Safety (lockable doors & windows, lockable cabinets for belongings)
- Bulonda (Cleanliness)
- Kujanika kwa zya kulya (Food availability)
- Mapenzi ajanika ciindi ca mwaka (mbuli ciluli kusweka, tuuka azyimwi zyintu)
  Seasonal problems (leaking roofs, insect problems, etc)

1c. Kuzwa nomwa kasika okuno, sena kuli nomwa kabana/kulala abamwi mubulo na a matress? Na ii, kamu pandulula mbo cakabede?

Did you have to share a bed or mattress during stay? If yes, please explain why

1d. Sena ciinzi ncomu bwene cijatikizya busena bwa kuyobweda ca kulya aa kwa kujikila?

What have you observed about the spaces for food storage and cooking?

i. Na kunyina kwa kujikila, sena bayobweda a kujikila kuli ca kulya?
   If no cooking space, where do women cook and store food?

1e. Sena ncinzi ncomu bwene akwendelezegwa kwa ng'anda yaku lindilila?

What have you observed about the ***management of the MWH***?

i. Sena kuli muntu wamu cooko naa mubelesi waa acibadela ugwasilizya kwendelezya ng'anda eyi?
   Is there either a community member or health facility staff who helps with the operation of this MWH?
ii. Sena kuli ulibonya kuba mwendelezyi ngomwaka swanya ciindi ni mwaka sika?
   Does anyone seem to be 'in charge' who you met with when you came to stay at the MWH?

1f. Kuti na kuli ciyandika kubambulula, sena mulizi baelede kuambilwa?
If there are ***maintenance concerns about the MWH***, do you know where to go to get this addressed?

1g. Sena mulizi bakuambila kuti naa kwajanika penzi ku ng'anda yakulindilila?
Who would you go to if there was a ***problem at the MWH***?

1h. Sena ciinzi ncomu bwene a lukamantano akati ka cibadela ang'anda ya kulindilila?
What have you observed about the ***linkages between the MWH and the hospital***?

i. Kuzwa ciindi no mwaka sika kung'anda ya kulindilila, sena kuli nobaka sika bacibadela kumilanganya?
During your stay have hospital staff come to shelter to check on women?

ii. Sena kuli noba inka kucibadela kupimwa mada bakaintu balindilila?
Do waiting women attend ANC check-ups at the hospital? How often?

1i. Zyintu nzyi zikonzya kuleta buyumu-yumu ku mu kaintu kuti abelesye ng'anda yakulindilila?
What things make it ***hard for a woman to use the MWH***?

Probe for:

- Bubotu bwa ng'anda yakulindilila (kukala kuka nyina ziyoosya, kukala kabotu, Tunsiya-nsiya, mapenzi ambi)
  Quality of MWH (safety, comfort, cultural appropriateness, other problems)
- Myulo (Costs)
- Cakulya akwa kujikila (Food or cooking space)
- Mboba mulanganya ku cibadela (Perceived quality of care at the health facility)
- Mizezo yaba mukwashi (Family member opinions)
- Milimo imbi ya ang'anda (Other responsibilities at home)
- Ciindi ca mwaka (Season)

1j. Zyintu nzyi zikonzya kuubya-ubya kumu kaintu kuti abelesye ng'anda yakulindilila?
What things make it ***easy for a woman to use the mothers' shelter***?

Probe for:

- Bubotu bwa ng'anda yakulindilila (kukala kuka nyina ziyoosya, kukala kabotu, Tunsiya-nsiya, mapenzi ambi)
  Quality of MWH (safety, comfort, cultural appropriateness, other problems)
- Myulo (Costs)
- Cakulya akwa kujikila (Food or cooking space)
- Mboba mulanganya ku cibadela (Perceived quality of care at the health facility)

- Mizezo yaba mukwashi (Family member opinions)
- Milimo imbi ya ang'anda (Other responsibilities at home)
- Ciindi ca mwaka (Season)

1k. Nkaambo nzi cimbi comwa salila kubola ku ng'anda yaba makaintu?

What are some of the ***reasons you chose to come*** to wait at a mothers' shelter at this hospital?

- Sena mwaka ambilwa kucipimo camada kuti muboole ku cibadela eci
  Were you referred from **ANC** to come to this hospital?
- Sena mwaka ambilwa kucipimo cabatumbu a a bana kuti mubole ku cibadela?
  Were you referred from **PNC** to come to this hospital?
- Ncoci badela cili afwafwi ambabo
  Is the closest health facility for them?
- Luzyibo ndondijisi acibadela eci naa ang'anda yaba makaintu?
  Was your perception of the MWH a factor?
- Sena mwaka zyiba buti ng'anda yaba makaintu eyi?
  How did you know about this MWH?

1l. Sena eyii njen'ganda njo mwakala ya kusanguna ada eyii?

Was this this ***first MWH*** you have stayed at for this pregnancy?

i. Na pepe, ninzi ncomwa zwida kuko?
   If no, why did you move?

Theme 2: Community Ownership and Sustainability of Mothers' Shelters

2a. Bantu bamu cooko bagwasilizya buti kuli zyakubamba kabotu ng'anda yabama tumbu na mayake kuci badela cenu?

How does the ***community contribute towards maintenance/upkeep*** of the MWH at your health facility?

Probe for:

- Kugwasilizya mali (Cash contributions)
- Kusanga cakulya (Food contributions)
- Kubeleka (Kukwapa, kulima, Kupyanga) (In-kind labour (slashing, weeding, sweeping)
- Kubambilila (Maintenance)
- Kusnga kulikonse (Any other contributions?)

2b. Sena mwaka badela naa kupa cili coonse kuti mukale mu ng'anda yaba makaintu eyi? Naa mbobo, ndalomba mupandulule

Did you have to ***pay or contribute*** anything in kind to stay at this mothers' shelter? Explain.

Theme 3: Barriers and facilitators to facility delivery

3a. Ninzi ncomwaka salila ku tumbukila ku cibadela eci kutali ku ng'anda?

Why did you choose to ***deliver at this hospital instead of at home***?

Probe for:

- Kutumwa abasikupima mada (Being referred by ANC)
- Misinzo azya kwendela (kamupandulula) (Distance/transport (ask for specifics))
- Myulo (kamu pandulula) (Costs (ask for specifics))
- Ciindi ca mwaka (kamu pandulula) (Seasons (ask for specifics))
- Milimo ya ang' anda (Household responsibilities)
- Kuzumizyigwa na kugwasilizigwa kumu kwasyi (Permission or support from family members)
- Mbociboneka cibadela aku langanizyigwa kabotu
  Perceptions of health facility and quality of care
- Mboba bona nseba yaba simada abana (Perceptions of pregnant women's or child's health risk)
- Twakushoma-shoma tutali kabotu tujatikizya kutumbukila ku ng'anda na ku cibaddela
  Myths and misconceptions about home or facility delivery

3b. Sena ninzi ncomwa salila ku tumbukila ku cibadela eci kwiinda ku kabadela kanini?

Why did you choose to ***deliver at this hospital instead of at rural health facility***?

Probe for:

- Kutumwa abasikupima mada (Being referred by ANC)
- Misinzo azya kwendela (kamupandulula) (Distance/transport (ask for specifics))
- Myulo (kamu pandulula) (Costs (ask for specifics))
- Ciindi ca mwaka (kamu pandulula) (Seasons (ask for specifics))
- Milimo ya ang' anda (Household responsibilities)
- Kuzumizyigwa na kugwasilizigwa kumu kwasyi (Permission or support from family members)
- Mbociboneka cibadela aku langanizyigwa kabotu
  Perceptions of health facility and quality of care
- Mboba bona nseba yaba simada abana (Perceptions of pregnant women's or child's health risk)
- Twakushoma-shoma tutali kabotu tujatikizya kutumbukila ku ng'anda na ku cibaddela
  Myths and misconceptions about home or facility delivery

3c. Mu mizezo yenu, ing'anda yabama kaintu (na busena bwaku lindilila) sena ilakulwaizya bamamama/bamukwashi kuba amuzezo waku tumbulila kucibadela? Ino kai?

In your opinion, does a mothers' shelter ***influence a woman/family's decision*** to deliver at a facility? Why or why not?

Theme 4: Preparedness and Costs

4a. Mali asika buti ayandika kuti mukaintu atumbukile ku chibadela eci?

What ***costs*** do women have when ***delivering at this hospital***?

Probe for:

- Zyakubelesha ciindi ca kutumbuka mbuli ma plastics, ma gloves, tumbeli azyimwi zyintu (Birth supplies including plastic sheets, gloves, sterile blade, etc)
- Zyisani zya mwana (Baby clothes)
- Zyendelo (Travel)
- Zya kulya (Food)
- Zya kubadela ciindi ca kutumbuka, zya kubadela zyitali mumulao, kupa zyintu zyimwi zyitali mumali kuli ba situmbuka
  Payments for delivery, informal payments, in-kind contributions to birth attendant

4b. Ino zya budelizyi zyindene buti kuti mukaintu wa tumbukila ku ng'anda

How do these costs differ when a woman ***delivers at home***?

Probe for:

- Zyakubelesha ciindi ca kutumbuka mbuli ma plastics, ma gloves, tumbeli azyimwi zyintu (Birth supplies including plastic sheets, gloves, sterile blade, etc)
- Zyisani zya mwana (Baby clothes)
- Zyendelo (Travel)
- Zya kulya (Food)
- Zya kubadela ciindi ca kutumbuka, zya kubadela zyitali mumulao, kupa zyintu zyimwi zyitali mumali kuli ba situmbuka
  Payments for delivery, informal payments, in-kind contributions to birth attendant

4c. Ino zyindene buti kuti mukaintu wa tumbukila ku kabadela kanini

How do these costs differ when a woman ***delivers at a rural health centre***?

Probe for:

- Zyakubelesha ciindi ca kutumbuka mbuli ma plastics, ma gloves, tumbeli azyimwi zyintu (Birth supplies including plastic sheets, gloves, sterile blade, etc)
- Zyisani zya mwana (Baby clothes)
- Zyendelo (Travel)
- Zya kulya (Food)
- Zya kubadela ciindi ca kutumbuka, zya kubadela zyitali mumulao, kupa zyintu zyimwi zyitali mumali kuli ba situmbuka
  Payments for delivery, informal payments, in-kind contributions to birth attendant

4d. Sena bama kaintu basowa mali manji buti kuula zyintu zyo babelesya ciindi noba tumbuka? Inoba zyuula kuli?

How much do women normally ***spend on birth supplies***? Where do women purchased them?

Probe for:

- Ma plastics (Plastic sheet)
- Ma gloves (Gloves)
- Mbeli (Sterile blade/razor)
- Tu pegs twa mukombo (Clamps)
- Zyimwi zyintu? (Anything else?)

4f. Sena bama kaintu ba zyuula kuli ezyi zisani?

Where do women normally ***purchase baby clothes***?

4h. Zyendelo nzi zyoba belesya bama kaintu kuunka ku cibadela kuyo tumbuka?

What ***modes of transport*** do women use to get to the hospital for delivery?

4k. Mali manji buti bamakaintu ngoba belesya kuula zya kulya ciindo nobali ku ng'anda yaku lindilila (kunze kwa zya kulya zyobaletelezya kuzwa ku ng'anda)

How much do women normally ***spend on food*** for during their stay at the MWH? (Separate from what they share with home)

4l. Sena mwakali yobwede mali akwendela kusika ku cibadela eci aa kulibambila ku tumbuka kuno? Did you ***save any money*** for the costs of traveling to the hospital and in preparation for delivery here?

i. Mwaka yobola buti? (How did you save?)

4m. Mbuyumu-yumu nzi bujanika muku yobola mali akubelesya kuli bambila kutumbuka?

What are the main ***challenges associated with saving money*** for delivery?

Probe with:

- Zyiyandika zimwi (tenda, kuciswa, zyi yandika maningi mu buumi)
  Competing needs (accidents, illness, basic needs)
- Mwaku yobweda mali kabotu (Safety of storing money)

This is the end of the Focus Group. Please thank the participants for their time.

Instrument ID: Form K5

The MAHMAZ Project

Focus Group Discussion Guide - ENGLISH/NYANJA

Target Audience:

1. *Pregnant or recently delivered (in the past 30 days) women who have been at CEmONC MWH for at least 1 night*
2. *Women >=15 years at last birthday*
3. *Has not previously participated in a FGD in the same year at another site or in another category*

Short screen:

Criteria	Response	Comments
SS1. Is the woman currently pregnant or recently delivered (in the past 30 days) and has stayed at least 1 night at the MWH?	Yes (1)<br>No (0)	If (0), do not proceed. Woman is not eligible.
SS2. Is the woman >=15 years old?	Yes (1)<br>No (0)	If (0), do not proceed. Woman is not eligible.
SS3. Has the woman previously participated in a focus group in the same year at the same or another site or other categories?	Yes (1)<br>No (0)	If (1), do not proceed. Woman is not eligible.

Instructions for the interviewer

Step 1: Informed consent: Ask each participant for a few minutes of their time. Introduce yourself and the study. Begin the informed consent process as per the training.

Was informed consent obtained and documented for each participant before the start of the FGD?

YES ______ (proceed with roster and FGD)

NO ______ (STOP! Thank the participant for their time but ask any who did not agree to leave. Then proceed with the FGD.)

***Interviewer**: Read the following statement to your FGD participants.*

"Zikomo po vomela kutenga ko mbali kuma funso aya. Zina langa ndine______. Ni zami funsani mafunso. Munzanga______ a zambo lemba zamene muza Kamba. Tiza recorda iyi mbali yamene ibwela.

Tifuno ziba maganizo yanu pa nyumba ya azimai naku kwanisa kuyenda maternity ku cipatala camu dela lanu. Munkale omasuka kutiuza zoonze zamane mutima unga kondwele kutiuza. Kumi kumbukisani chabe, nipempa musapeleke zamene munvele olo zikambiwa pano kuma gulu ena. Elo mufunika kuziba kuti simufinika kukamba zili zoonse zamene mutima wanu siumi vomekeza. Siti zafaka pamodzi zamene muzakamba na mayanko yanu. Kulibe mayanko ya bwino olo yoipa, nipempa munkale bokulupilika nakutiuza zonadi zochokela kuli imwe namu dela lanu. Ndimwe okonzeka kuti tiyambe?"

Step 2: *Please complete the roster table for each participant as they sign in. Verify eligibility. This form will have an ID letter for each participant. Make sure that the note taker has the correct ID letter recorded on his/her notes prior to beginning and that participants have their correct "Letter Label".*

Step 3: *Proceed to the FGD. Start by reiterating the importance of confidentiality within the group. As you ask the questions, please probe to obtain as much information as possible for each question.*

Facilitator name ____________________ **Note-taker Name ____________________**

1. **FGD Date (DD/MM/YYY) ________________**
2. **Health Facility Name ________________**
3. **Health Facility ID ________________**
4. **District Name ________________**
5. **Province Name ________________**
6. **Time start (HH:MM) ________** **Time finish (HH:MM) ________**
7. **Supervisor initials __________**

INSTRUMENT K5:
Focus Group Discussion Guide for CEmONC Maternity Waiting Home

GROUP ID

Demographics: Pregnant/ Recently Delivered Women

ID	Age at last birthday	Pregnant or recently delivered<br><br>Pregnant (1)<br><br>Recently delivered (2)	If pregnant, how many months is the pregnancy?	If recently delivered, age of baby months (if <1 month, put 0)	Prev FGD this Round?<br><br>Yes (1)<br>No (0)<br><br>If 1, person not eligible to participate	Eligibility confirmed? 1/0<br><br>If 1, proceed with the rest of the columns<br><br>If 0, stop and thank the woman for her time	Gender<br>Male (1)<br>Female (2)	Marital status:<br><br>Married/ cohabiting (1)<br><br>Divorced (2)<br><br>Separated (3)<br><br>Widowed (4)<br><br>Never married (5)	What is the highest grade you completed?<br><br>If < grade 1, put 0. If > grade 12, put 13.	How many live, biological children do you have?	Total # of pregnancies including current one	For your most recent pregnancy, where did/will you deliver?<br>In a home (1)<br>Health facility (2)<br>Hospital (3)<br>Other (4)<br>(specify)	What HF are you closest to?	Travel time to this MS (include units – minutes or hours)
**A**							**2**							
**B**							**2**							
**C**							**2**							
**D**							**2**							

INSTRUMENT K5:
Focus Group Discussion Guide for CEmONC Maternity Waiting Home

GROUP ID

ID	Age at last birthday	Pregnant or recently delivered<br><br>Pregnant (1)<br><br>Recently delivered (2)	If pregnant, how many months is the pregnancy?	If recently delivered, age of baby months (if <1 month, put 0)	Prev FGD this Round?<br><br>Yes (1)<br>No (0)<br><br>If 1, person not eligible to participate	Eligibility confirmed? 1/0<br><br>If 1, proceed with the rest of the columns<br><br>If 0, stop and thank the woman for her time	Gender<br>Male (1)<br>Female (2)	Marital status:<br><br>Married/ cohabiting (1)<br><br>Divorced (2)<br><br>Separated (3)<br><br>Widowed (4)<br><br>Never married (5)	What is the highest grade you completed?<br><br>If < grade 1, put 0. If > grade 12, put 13.	How many live, biological children do you have?	Total # of pregnancies including current one	For your most recent pregnancy, where did/will you deliver?<br>In a home (1)<br>Health facility (2)<br>Hospital (3)<br>Other (4) (specify)	What HF are you closest to?	Travel time to this MS (include units – minutes or hours)
E							2							
F							2							
G							2							
H							2							

Theme 1: Awareness, Utilization and Quality of the Maternity Waiting Homes

1a. Kambani zamene muziba pa nyumba ya cidikilo?

Tell me what you know ***about MWHs in general.***

i. Ili na cinto ya bwanji? (What are they for?)
ii. Nibandani baenela kunkala mo? (Who can stay there?)

1b. Ni ciani camene mwaona kuli mwamene ilili nyumba ya cidikilo pa cipatala ici?

What have you observed about the ***quality of the MWH*** at this hospital?

Probe for:

- Kunkala bwino (kamangidwe, zo pezeka mo, vo yambata, ma mosquito nets, va miambo)

  Comfort (Structure, furnishings, bedding, bed nets, cultural appropriateness)
- Kupaka kwa bantu (Crowding)
- Kunkala palibe zo yofia (katundu wanu uli bwino, bantu bankala cabe bwino)

  Safety (belongings safe, people staying there)
- Udongo (Cleanliness)
- Kupezeka kwa vo kudya (Food availability)
- Mabvuto yapa ntawi mu caka (mitenge yamene iloka, mabvuto ya tudoyo, navina)
  Seasonal problems (leaking roofs, insect problems, etc)

1c. Kuchokela pamene muna bwela kuno, kuli pamene muna gona pa bed kapena pa matress na muntu wina? Ngati ni telo, fotokozani

Did you have to share a bed or mattress during stay? If yes, please explain why

1d. Kodi ni ciani camene munaona pamalo yo pikila na ku sungila va kudya?

What have you observed about the spaces for food storage and cooking?

i. Ngati kulibe malo yopikila, apikila no sungila vo kudya kuti?
   If no cooking space, where do women cook and store food?

1e. Kodi ni ciani camene munaona pa kaendelezedwe ka nyumba ya cidikilo?

What have you observed about the ***management of the MWH***?

i. Kodi kuli wamu munzi kapena wa cipatala wamene ama tandizila kaendelezedwe ka nyumba ya cidikilo?
   Is there either a community member or health facility staff who helps with the operation of this MWH?

ii. Kodi kuli bamene munaona kuti baendeleza nyumba ya cidikilo bamene muna peza pamene muna bwela?
Does anyone seem to be 'in charge' who you met with when you came to stay at the MWH?

1f. Ngati kuli vokonza vamene vifunika pa nyumba ya cidikilo, kodi muziba bo uza kuti bavi konze?
If there are ***maintenance concerns about the MWH***, do you know where to go to get this addressed?

1g. Ngati kuli mabvuto ku nyumba ya cidikilo, kodi muziba vo uza?
Who would you go to if there was a ***problem at the MS***?

1h. Kodi ni ciani camene mwaona pa cigwilizano pakati ka cipatala na nyumba ya cidikilo?
What have you observed about the ***linkages between the MWH and the hospital***?

i. Pa ntawi yamene mwankala kuno kodi ba cipatala bana bwela ko kuona azimai kuno?
During your stay have hospital staff come to shelter to check on women?

ii. Kodi azimai amene adikila ameyenda ku cipatala ku cipimo ca mimba? Ka ngati?
Do waiting women attend ANC check-ups at the hospital? How often?

1i. Nivicani vinga nkale vo limba kuli muzimai kuti asebenzese nyumba yo dikila mo?
What things make it ***hard for a woman to use the MWH***?
Probe for: (ask about each bullet separately)

- Ubwino wa yumba yo dikila mo (kunkala kopanda zo yofya, kunkala bwino, ku lingana na myambo, na vina vabwino, etc
  Quality of MWH (safety, comfort, cultural appropriateness, other problems)
- Ndalama (Costs)
- Ca kudya na ko pikila (Food or cooking space)
- Kasamalidwe ka bwino ka ku cipatala (Perceived quality of care at the health facility)
- Maganizo ya banja (Family member opinions)
- Cinto zina za ku nyumba (Other responsibilities at home)
- Ntawi pa caka (Season)

1j. Znivicani vinga nkale vo pepuka kuli muzimai kuti asebenzese nyumba yo dikila mo?
What things make it ***easy for a woman to use the MWH***?
Probe for: (ask about each bullet separately)

- Ubwino wa yumba yo dikila mo (kunkala kopanda zo yofya, kunkala bwino, ku lingana na myambo, na vina vabwino, etc
  Quality of MWH (safety, comfort, cultural appropriateness, other problems)
- Ndalama (Costs)

- Ca kudya na ko pikila (Food or cooking space)
- Kasamalidwe ka bwino ka ku cipatala (Perceived quality of care at the health facility)
- Maganizo ya banja (Family member opinions)
- Cinto zina za ku nyumba (Other responsibilities at home)
- Ntawi pa caka (Season)

1k. Kodi ni ciani muna sankila kudikila ku cidikilo capa cipatala pano?

What are some of the ***reasons you chose to come*** to wait at a MWH at this hospital?

- Kodi muna tumiwa kuchokela ku cipimo ca mimba kuti mu bwele ku cipatala ici?
  Were you referred from **ANC** to come to this hospital?
- Kodi muna tumiwa kuchokela ku cipimo ca bamai na mwana kuti mu bwele ku cipatala ici?
  Were you referred from **PNC** to come to this hospital?
- Ndiye cipatala cili pafupi
  Is the closest health facility for them?
- Kodi maganizo ya nyumba ya cidikilo yana liko?
  Was your perception of the MS a factor?
- Kodi muna ziba bwanji za nyumba ya cidikilo?
  How did you know about this Maternity Waiting Home?

1l. Kodi ndiye nyumba ya cidikilo yo yamba yamene mwa nkala mo pa mimba iyi?

Was this this ***first MWH*** you have stayed at for this pregnancy?

i. Ngati sitelo ni ciani muna bwela kuno?
   If no, why did you move?

Theme 2: Community Ownership and Sustainability of Maternity Waiting Homes

2a. Bantu bamu dela lanu batandizila bwanji kuli zama sungidwe ya nyumba yo dikila ku cipatala canu??

How does the ***community contribute towards maintenance/upkeep*** of MWH at your health facility?

Probe for issues related to:

- Kutandizila ndalama (Cash contributions)
- Kupeleka mphaso ya za kudya (Food contributions)
- Kusewenza monga (ku kwapa ku pyanga, ku kusakulia na zina zace)
  (In-kind labor (slashing, weeding, sweeping, etc))
- Kusamalila (Maintenance)
- Kupeleka mphaso iliyonse (Any other contributions?)

2b. Kodi muna lipila kapena kupasa ciliconse kuti munkale mu nyumba ya cidikilo? Fotokozani

Did you have to ***pay or contribute*** anything in kind to stay at this MWH? Explain.

Theme 3: Barriers and facilitators to facility delivery

3a. Kodi ni ciani camene mwa sankila ku papila ku cipatala kusiya ku nyumba?

Why did you choose to ***deliver at this hospital instead of at home***?

Probe for: (ask about each bullet separately, do not ask all at once!)

- Kutumidwa kuchokela ku chipimo cha mimba (Being referred by ANC)
- Mitunda na voyendela(masulani) (Distance/transport (ask for specifics))
- Mitengo (masulani) (Costs (ask for specifics))
- Ntawi ya caka (Masulani) (Seasons (ask for specifics))
- Ncinto zapa nyumba (Household responsibilities)
- Kuvomekezedwa kapena kutandizidwa na banja
  Permission or support from family members
- Maonekedwe achipatala na mwamene akusungilani
  Perceptions of health facility and quality of care
- Mwamemena baonela mabvuto yamene yanga pezeke pa umoyo wa azimai ali pakati na bana (Perceptions of pregnant women's or child's health risk)
- Va miambo na vina vokululupila po balila ku nyumba kapena ku cipatala
  Myths and misconceptions about home or facility delivery

3b. Kodi ni ciani muna sankila ku papilla ku cipatala ici kusiya cipatala cing'ono?

Why did you choose to ***deliver at this hospital instead of at rural health facility***?

Probe for: (ask about each bullet separately)

- Kutumidwa kuchokela ku chipimo cha mimba (Being referred by ANC)
- Mitunda na voyendela(masulani) (Distance/transport (ask for specifics))
- Mitengo (masulani) (Costs (ask for specifics))
- Ntawi ya caka (Masulani) (Seasons (ask for specifics))
- Ncinto zapa nyumba (Household responsibilities)
- Kuvomekezedwa kapena kutandizidwa na banja
  Permission or support from family members
- Maonekedwe achipatala na mwamene akusungilani
  Perceptions of health facility and quality of care
- Mwamemena baonela mabvuto yamene yanga pezeke pa umoyo wa azimai ali pakati na bana (Perceptions of pregnant women's or child's health risk)
- Va miambo na vina vokululupila po balila ku nyumba kapena ku cipatala
  Myths and misconceptions about home or facility delivery

3c. Muma ganizo yanu, kodi nyumba yo dikila ima pasa ganizo kuli muzimai ali napakati/na banja kuti ankale ndi maganizo yo papilla kuchipatala? fotokozani?

In your opinion, does a Maternity Waiting Home ***influence a woman/family's decision*** to deliver at a facility? Why or why not?

Theme 4: Preparedness and Costs

4a. Kodi Muzimai asebenzesa ndalama za mbili bwanji ku papilla pa cipatala ici?

What ***costs*** do women have when ***delivering at this hospital***?

Probe for:

- Vo sebenzesa popapa monga ma plastics, ma gloves, kaleza navina vache
  Birth supplies including plastic sheets, gloves, sterile blade, etc
- Vo vala va mwana (Baby clothes)
- Vo yendela (Travel)
- Vo kudya (Food)
- Ko gona (Accommodation)
- Volipila pa ntawi yo papa, malipilo osa zibika na lamulo, vopasa vina osati ndalama
  Payments for delivery, informal payments, in-kind donations

4b. Ndalama isiyana bwanji ngati muzimai apapila ku nyumba?

How do these costs differ when a woman ***delivers at home***?

Probe for:

- Vo sebenzesa popapa monga ma plastics, ma gloves, kaleza navina vache
  Birth supplies including plastic sheets, gloves, sterile blade, etc
- Vo vala va mwana (Baby clothes)
- Vo yendela (Travel)
- Vo kudya (Food)
- Ko gona (Accommodation)
- Volipila pa ntawi yo papa, malipilo osa zibika na lamulo, vopasa vina osati ndalama
  Payments for delivery, informal payments, in-kind donations

4c. Kodi ndalama isiyana bwanji ngati muzimai apapila ku cipatala cing'ono?

How do these costs differ when a woman ***delivers at a rural health centre***?

Probe for:

- Vo sebenzesa popapa monga ma plastics, ma gloves, kaleza navina vache
  Birth supplies including plastic sheets, gloves, sterile blade, etc
- Vo vala va mwana (Baby clothes)
- Vo yendela (Travel)
- Vo kudya (Food)

- Ko gona (Accommodation)
- Volipila pa ntawi yo papa, malipilo osa zibika na lamulo, vopasa vina osati ndalama
  Payments for delivery, informal payments, in-kind donations

4d. Kodi azimai ama taya ndalama zambili bwanji pazo sebenzesa kupapa? Kodi ama zigula kuti?

How much do women normally ***spend on birth supplies***? Where do women purchased them?

Probe for:

- Ma Plastics (Plastic sheet)
- Ma Gloves (Gloves)
- Tu leza (Sterile blade/razor)
- Tuma pegs twa pa mukombo (Clamps)
- Na zina zache? (Anything else?)

4f. Kodi azimai amazigula kuti vovala vamwana?

Where do women normally ***purchase baby clothes***?

4h. Kodi bazimai Bama sebenzensa viyendelo va bwanji po yenda kupapa ku cipatala?

What ***modes of transport*** do women use to get to the health facility for delivery?

4k. Ni ndalama yambili bwanji yamene muzimai anga sebenzese ku gula va kudya pa ntawi yamene ali kunyumba ya cidikikilo (kuchosela ko vamene babwela navo kuchoka ku nyumba)

How much do women normally ***spend on food*** for during their stay at the MS? (Separate from what they share with home)

4l. Kodi muna sunga ndalama zo yendela ku cipatala nazo konzekela kupapila kuno?

Did you ***save any money*** for the costs of traveling to the hospital and in preparation for delivery here?

i. Muna sunga bwanji? (How did you save?)

4m. Kodi ni mabvuto yotani yamene yapezeka po sunga ndalama yo sebenzesa po papa?

What are the main ***challenges associated with saving money*** for delivery?

Probe with:

- Zina zace zo funikila(ngozi, matenda, zo funikila maningi mu umoyo)
  Competing needs (accidents, illness, basic needs)
- Mo sungila bwino ndalama (Safety of storing money)

This is the end of the Focus Group. Please thank the participants for their time.
